# Extracellular Vesicle Transmission of Chemoresistance to Ovarian Cancer Cells Is Associated with Hypoxia-Induced Expression of Glycolytic Pathway Proteins, and Prediction of Epithelial Ovarian Cancer Disease Recurrence

**DOI:** 10.3390/cancers13143388

**Published:** 2021-07-06

**Authors:** Mona Alharbi, Andrew Lai, Shayna Sharma, Priyakshi Kalita-de Croft, Nihar Godbole, America Campos, Dominic Guanzon, Alexis Salas-Burgos, Flavio Carrion, Felipe A. Zuñiga, Lewis Perrin, Yaowu He, Tanja Pejovic, Carmen Winters, Terry Morgan, John D. Hooper, Gregory E. Rice, Carlos Salomon

**Affiliations:** 1 Exosome Biology Laboratory, Centre for Clinical Diagnostics, University of Queensland Centre for Clinical Research, Royal Brisbane and Women’s Hospital, The University of Queensland, Brisbane, QLD 4029, Australia; mona.alharbi@uq.net.au (M.A.); a.lai@uq.edu.au (A.L.); s.sharma@uq.edu.au (S.S.); n.godbole@uq.net.au (N.G.); america.camposg@gmail.com (A.C.); d.guanzon@uq.edu.au (D.G.); g.rice@uq.edu.au (G.E.R.); 2 Department of Biochemistry, College of Science, King Saud University, Riyadh 11451, Saudi Arabia; 3 Faculty of Medicine, University of Queensland Centre for Clinical Research, Royal Brisbane and Women’s Hospital, The University of Queensland, Herston, QLD 4029, Australia; p.kalita@uq.edu.au; 4 Department of Pharmacology, Faculty of Biological Sciences, University of Concepción, Concepción 4030000, Chile; alsalas@udec.cl; 5 Departamento de Investigación, Postgrado y Educación Continua (DIPEC), Facultad de Ciencias de la Salud, Universidad Pedro de Valdivia, Santiago 8320000, Chile; flavio.carrion@upv.cl; 6 Department of Clinical Biochemistry and Immunology, Faculty of Pharmacy, University of Concepción, Concepción 4030000, Chile; fzuniga@udec.cl; 7 Mater Research Institute, University of Queensland, Translational Research Institute, Woolloongabba, QLD 4102, Australia; lewisperrin@mc.mater.org.au (L.P.); yaowu.he@mater.uq.edu.au (Y.H.); john.hooper@mater.uq.edu.au (J.D.H.); 8 Departments of Obstetrics and Gynecology and Pathology, Oregon Health & Science University, Portland, OR 97239, USA; pejovict@ohsu.edu (T.P.); wintersc@ohsu.edu (C.W.); morgante@ohsu.edu (T.M.); 9 Centro de Investigación e Innovación Biomédica, Universidad de los Andes, Santiago 8320000, Chile

**Keywords:** ovarian cancer, hypoxia, exosomes, extracellular vesicles

## Abstract

**Simple Summary:**

Ovarian cancer is one of the most lethal cancers affecting women worldwide. Its high mortality rate is often attributed to the non-specific nature of early symptoms of the disease. Developing a better understanding of the disease progression and identifying clinically useful biomarkers that aid in clinical management are requisite to reducing the mortality rate of ovarian cancer. Reduced oxygen tension (i.e., hypoxia) is not only a characteristic of solid tumors but may also enhance the metastatic capacity of tumors by inducing the release of tumor growth promoting factors. Recently, it has been proposed that small tumor-derived extracellular vesicles (sEVs) facilitate cancer progression. In this study, we established that sEVs produced under low oxygen tension induce a metabolic switch in ovarian cancer cells associated with changes in glycolytic pathway proteins that promote resistance to carboplatin. Significantly, we identified a suite of sEV-associated glycolysis pathway proteins that are present in patients with ovarian cancer that can predict disease recurrence with over 90% accuracy.

**Abstract:**

Hypoxia is a key regulator of cancer progression and chemoresistance. Ambiguity remains about how cancer cells adapt to hypoxic microenvironments and transfer oncogenic factors to surrounding cells. In this study, we determined the effects of hypoxia on the bioactivity of sEVs in a panel of ovarian cancer (OvCar) cell lines. The data obtained demonstrate a varying degree of platinum resistance induced in OvCar cells when exposed to low oxygen tension (1% oxygen). Using quantitative mass spectrometry (Sequential Window Acquisition of All Theoretical Fragment Ion Mass Spectra, SWATH) and targeted multiple reaction monitoring (MRM), we identified a suite of proteins associated with glycolysis that change under hypoxic conditions in cells and sEVs. Interestingly, we identified a differential response to hypoxia in the OvCar cell lines and their secreted sEVs, highlighting the cells’ heterogeneity. Proteins are involved in metabolic reprogramming such as glycolysis, including putative hexokinase (HK), UDP-glucuronosyltransferase 1–6 (UD16), and 6-phosphogluconolactonase (6 PGL), and their presence correlates with the induction of platinum resistance. Furthermore, when normoxic cells were exposed to sEVs from hypoxic cells, platinum-resistance increased significantly (*p* < 0.05). Altered chemoresistance was associated with changes in glycolysis and fatty acid synthesis. Finally, sEVs isolated from a clinical cohort (*n* = 31) were also found to be enriched in glycolysis-pathway proteins, especially in patients with recurrent disease. These data support the hypothesis that hypoxia induces changes in sEVs composition and bioactivity that confers carboplatin resistance on target cells. Furthermore, we propose that the expression of sEV-associated glycolysis-pathway proteins is predictive of ovarian cancer recurrence and is of clinical utility in disease management.

## 1. Introduction

Ovarian cancer is the most lethal gynecological cancer [1]. Delayed diagnosis, high metastatic frequency, and resistance to chemotherapy contribute to the lethality of ovarian cancer [1]. Carboplatin, paclitaxel, or a combination of both are the first line chemotherapy treatments for ovarian cancer [2]. Survival rates following treatment are less than 20%, as ovarian cancer often recurs with chemoresistance, making this type of cancer extremely difficult to treat [2]. Carboplatin is a DNA-binding alkylating agent (non-specific cell phase) that displays response rates of ~70% in newly diagnosed patients with advanced disease [3,4]. In the presence of tissue hypoxia, the response to carboplatin is significantly reduced [5]. Hypoxia has emerged as a significant feature of the malignant tumor microenvironment and is considered a critical factor in promoting tumor metastasis and chemoresistance associated with poor prognosis [6,7].

Typically, hypoxic regions develop within tumors, as their growth cannot be fully supported by their vascular supply. This results in a spatial disorganization of tumor vascular networks to meet cell demand for oxygen and nutrition [8]. Disorganized and immature blood vascular networks can cause unstable blood flow and limit oxygen diffusion [9,10]. Under a hypoxic environment, cancer cells modulate their metabolic programming to meet their oxygen and nutrient requirements which accelerates cancer progression and increases cell survival and adaptation. For example, during hypoxic exposure, cancer cells induce the activity of glycolytic enzymes to convert glucose into lactate, to provide sufficient energy and biomass supporting cell survival and proliferation [11,12]. This process causes the cancer cells to secrete CO_2_ and carbonic acid (low pH) and thus limits the effectiveness of chemotherapeutic agents [13,14]. The acidic environment produced by hypoxic cells increases the activity of P-glycoprotein (Pgp) in cancer cells [15]. Pgp is an efflux pump protein that reduces the cytotoxicity and accumulation of chemo drugs in the cytosol [13].

Recent studies highlight the role of small extracellular vesicles (sEVs) in ovarian cancer progression [16,17,18]. sEVs are lipid bilayer-encapsulated vesicles that are naturally released from a wide range of cells including cancer cells [19]. sEVs are selectively packaged with bioactive molecules, including proteins and small non-coding RNA, that are secreted by exocytosis into biofluid compartments [20]. EVs modulate the activity of both proximal and distal cells to modify several biological processes such as angiogenesis [19], proliferation [21], and metabolism [22]. EVs signaling, thus, represents an integral pathway mediating cell to cell communication in many cancers, including ovarian cancer [16]. Small EVs like exosomes released from cancer cells exposed to low oxygen tension reduced apoptosis in target cells [23], suggesting that exosomes might be involved in chemotherapy resistance.

The available evidence suggests that the development of tumor resistance to chemotherapy is, in part, due to cell-cell transfer of effectors within the tumor microenvironment [24,25]. For example, hypoxia increases the release of exosomes from cancer cells, alters their cargo, and enhances cell migration and invasion in target cells [26]. Similarly, cells isolated from ascites obtained from patients with ovarian cancer and cultured under hypoxic conditions release sEVs enriched with STAT3 and FAS; oncogenic proteins can induce both cell migration (in vitro) and tumor invasion in vivo [27].

The aim of this study was to test the hypothesis that carboplatin-resistant cancer cells release sEV-associated proteins, under hypoxic conditions, that confer chemoresistance to recipient cells. This hypothesis was further tested by establishing the capacity of sEV-associated proteins, isolated from ovarian cancer patients, to predict chemo-resistance and disease recurrence.

## 2. Results

### 2.1. Hypoxia Induces Chemoresistance in Epithelial Ovarian Cancer Cells

**Figure 1:**
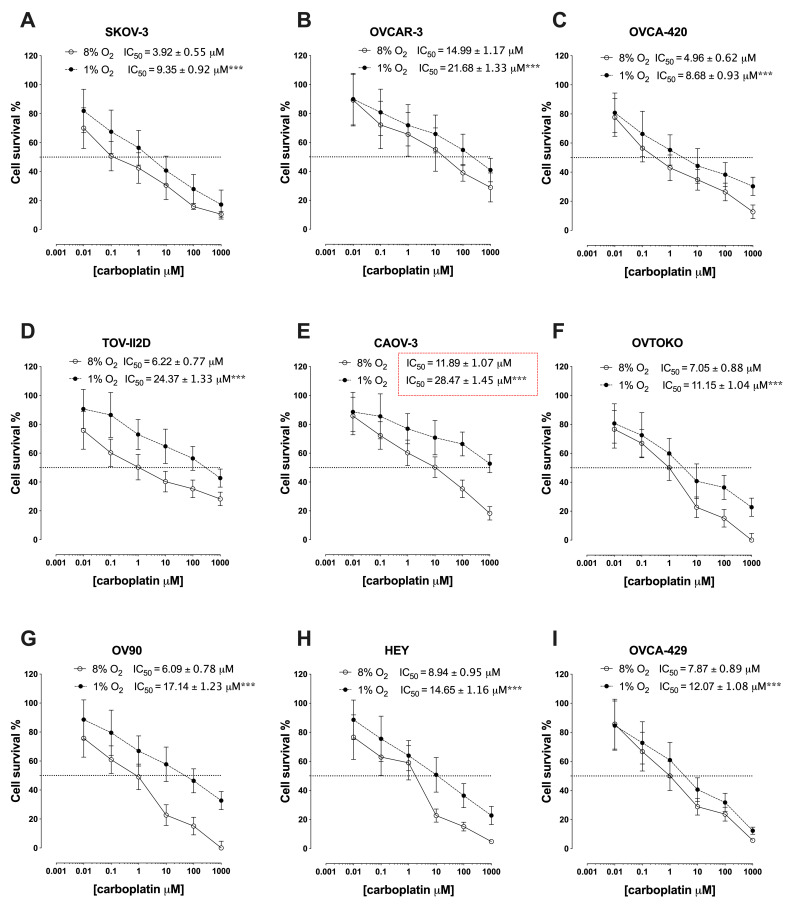
IC50 of a panel of epithelial ovarian cancer cells. The cells ((**A**–**I**): SKOV-3, OVCAR-3, OVCA-420, TOV-112D, CAOV-3, OVTOKO, OV-90, HEY, and OVCA-429) were exposed to either 1% O_2_ or 8% O_2_ and treated with carboplatin at different concentrations (0, 0.001 µM, 0.01 µM, 0.1 µM, 1 µM, 10 µM, 100 µM, and 1000 µM) in the presence of Caspase-3/7 reagent. The viability of the cells was assessed using the IncuCyte™ real-time cell-imaging system every 2 h for 72 h. The data are represented by the mean ± SEM (*n* = 6). The IC50 of all EOC cells in this study were higher under hypoxia than under normoxic in their parental cells. *** *p* < 0.0005 at 1% oxygen compared with 8% oxygen.

To investigate the effects of hypoxia on the response of ovarian cancer cells to the chemotherapy drug, carboplatin, a panel of nine epithelial ovarian cancer (EOC) cell lines were cultured under oxygen tensions that approximate those found in normal tissue (8% O_2)_ and hypoxic tumors (1% O_2_) [28]. Subsequently, cell viability in response to different concentrations of carboplatin [0.01, 0.1, 1, 10, 100, 1000 µM] was evaluated. CAOV-3 cells displayed the greatest resistance to carboplatin under 1% O_2_, followed by TOV-112D, OVCAR-3, OV90, HEY, OVCA-429, OVTOKO, SKOV-3, and OVCA-420 (Figure 1A–I). Under 8% O_2_, SKOV-3 and OVCA-420 were the most sensitive EOCs to carboplatin (IC_50_ = 3.9 ± 0.5 µM and 4.96 ± 8.68 µM, respectively). Under hypoxic conditions, CAOV-3 developed the highest resistance to carboplatin (IC_50_ = 28.47 ± 1.45 µM) and exhibited the most significant dose response curve shift, therefore, this cell line was selected for further characterization.

### 2.2. Hypoxia Induced Carboplatin-Resistance Is Characterized by Metabolic Pathway Dysregulation

**Figure 2:**
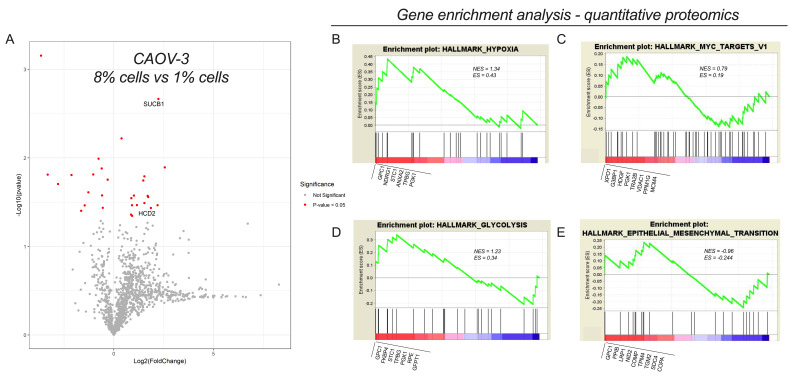
Quantitative proteomic analysis of CAOV-3 cells exposed to 1% O_2_ and 8% O_2_. (**A**). Volcano plot of the quantified proteins showing the differentially expressed proteins in the CAOV-3 cells exposed to 1% oxygen tension compared with cells exposed to 8% oxygen tension (upregulated on the right side and downregulated on the left side). 30 proteins were significantly expressed in the hypoxic cells (1% O_2_ CAOV-3) than in the normoxic cells. Gene set enrichment analysis (GSEA) of proteomic data illustrate four gene clusters that are enriched in the 1% O_2_ CAOV-3 cells compared cells at 8% O_2_ namely, (**B**) hypoxia, (**C**) epithelial–mesenchymal transition, (**D**) glycolysis, and (**E**) Myc target genes. The protein subset was evaluated using the normalized enrichment score (the green line). In the GSEA figures, the black vertical lines identify the positions where the members of a particular pathway appear in the ranked list of genes. The upregulated genes in red are localized on the left, while the downregulated proteins in blue are on the right.

Hypoxia may induce proteomic changes that control cell growth, activate the survival pathway of cells, mediate cell-cycle arrest, and promote cancer progression. We, therefore, investigated whether hypoxia-induced changes in carboplatin resistance in COVA-3 cells are associated with changes in the proteomic profile. Changes in protein abundance were determined by quantitative mass spectrometry analysis using a sequential window acquisition of all theoretical fragment ion spectra mass spectrometry (SWATH). Information-dependent acquisition (IDA) and SWATH profile were generated from CAOV-3 cultured at 8% and 1% O_2_ using independent samples (*n* = 6, per each group). The IDA library was used to identify peptide ions present in SWATH ion profiles. Proteins were identified and quantified by comparing SWATH-generated peptide ion profiles for each individual sample against the IDA library (PeakView). IDA of mass spectra from all individual samples was initially performed and identified 1233 total proteins and analyzed using IDA and SWATH. The variation in the relative abundance of proteins between cells (8% O_2_ or 1% O_2_) was established by comparison with the SWATH profile against the IDA library and presented as volcano plot (Figure 2A). A total of 30 proteins were significantly differentially expressed in cells that were cultured under hypoxic conditions when compared with normoxic cells (i.e., 8% O_2_) (Supplemental Table S1). From the MS/MS data, we identified a significant increase in the detoxification and ATP production enzymes: 3-hydroxyacyl-CoA dehydrogenase type-2 (HCD2) and Succinate—CoA ligase (SUCB1) (*p* = 0.037 and *p* = 0.002, respectively).

To gain further insights into the biological functions of the dysregulated proteins, MS/MS profile data were subjected to gene enrichment analysis (GSEA), revealing several gene sets to be significantly enriched in hypoxic cells compared with normoxic cells. This is demonstrated by the normalized enrichment score that is the primary statistic that determines whether a gene set is overrepresented in a ranked list of genes and has an FDR <0.01. GSEA showed an enrichment of proteins involved in hypoxia, epithelial mesenchymal transition, glycolysis, and MYC targets (Figure 2B–E). These results establish that hypoxia changes the proteomic profile of CAOV-3 cells, involved in the expression of the glycolysis signaling pathway and proteins that are important in the metabolic requirements associated with cellular response to chemotherapy.

### 2.3. Analysis of sEVs from CAOV-3 Cells

**Figure 3:**
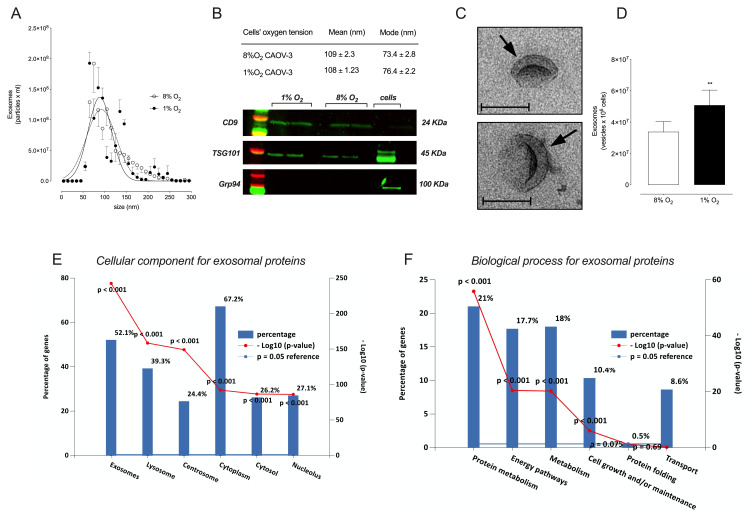
Isolation and characterization of 1% O_2_ and 8% O_2_ CAOV-3 derived sEVs. (**A**) Size distribution of sEVs isolated from 1% O_2_ and 8% O_2_ CAOV-3 derived sEVs determined using nanoparticle tracking analysis. (**B**) Representation of the mean and mode of the 1% O_2_ and 8% O_2_ and Western blot of exosomal protein markers CD9, and TSG101, and the negative control for both the 1% O_2_ sEVs and 8% O_2_ sEVs Grp94. (**C**) Transmission electron microscopy images of the isolated sEVs suggested the presence of vesicles. (**D**) Quantification of exosome particles from 1% O_2_ and 8% O_2_ sEVs presented as the number of particles/106 cells/48 h. (**E**) FunRich-based enrichment analysis of the exosome component from the SWATH analysis of sEVs. (**F**) FunRich-based enrichment analysis of the biological process of the exosome proteins. ** *p* < 0.005.

This study further investigated the effects of hypoxia on cellular protein abundance and if these changes are reflected in sEVs released from ovarian cancer cells. CAOV-3 cells were used because a significant switch in cell apoptosis in response to carboplatin in 1% oxygen compared with 8% oxygen was observed (i.e., from 11 to 28 µM, Figure 1). Extracellular vesicles were isolated from CAOV-3 cell-conditioned media and cultured at 8% and 1% O_2_ for 48 h. The size distribution of the vesicles is presented in Figure 3A. No significant differences were observed in the size distribution of the EVs from cells cultured with 8% O_2_ and those cultured with 1% O_2_. The EV preparations contained vesicles ~100 nm in diameter and were consistent with the size range and morphology of sEVs (Figure 3A,C). sEVs were positive for CD9 and TSG101 (Figure 3B and Figure S4), proteins associated with vesicles from an endocytic origin-like sEVs. The EV preparation was negative for Grp94 (Figure 3B and Figure S4); an endoplasmic reticulum marker used to establish the purity of EV isolates. The release of EVs from cells incubated under hypoxic conditions was 2-fold greater (*p* < 0.05) than that observed under 8% O_2_ (Figure 3D).

The proteomic profile of sEVs from the CAOV-3 cultured at 8% and 1% O_2_ was determined using the IDA of all individual sEV samples as well as SWATH. A total of 1233 EV-associated proteins were identified and subjected to functional enrichment analysis using FunRich (http://www.funrich.org (accessed on 20 December 2019)) software to further elucidate their putative biological relevance. Proteins were characterized as cytoplasmic (67.3%) and exosomal proteins (52.2%), respectively (Figure 3E). Functionally, these proteins were found to be involved in protein metabolism (21%), metabolism (18%), and energy pathways (17%) (Figure 3F). A total of 35 proteins from the 100 exosome-associated proteins present in the ExoCarta database were identified in the sEV samples. Overall, these results confirmed the reproducibility of the present method to enrich a specific type of EVs, sEVs. sEVs were defined by the enrichment of proteins associated with sEVs (i.e., CD9, and TSG101) as well as a lack of Grp94 (negative control); size distribution of ~100 nm (within the size of the sEVs); and spherical morphology. SWATH analysis matched the sEVs database and revealed that an enriched population of sEVs was isolated in the present study.

### 2.4. sEVs Protein Cargo Is Altered by Oxygen Tension and Is Selectively Enriched for Chemoresistant Factors

**Figure 4:**
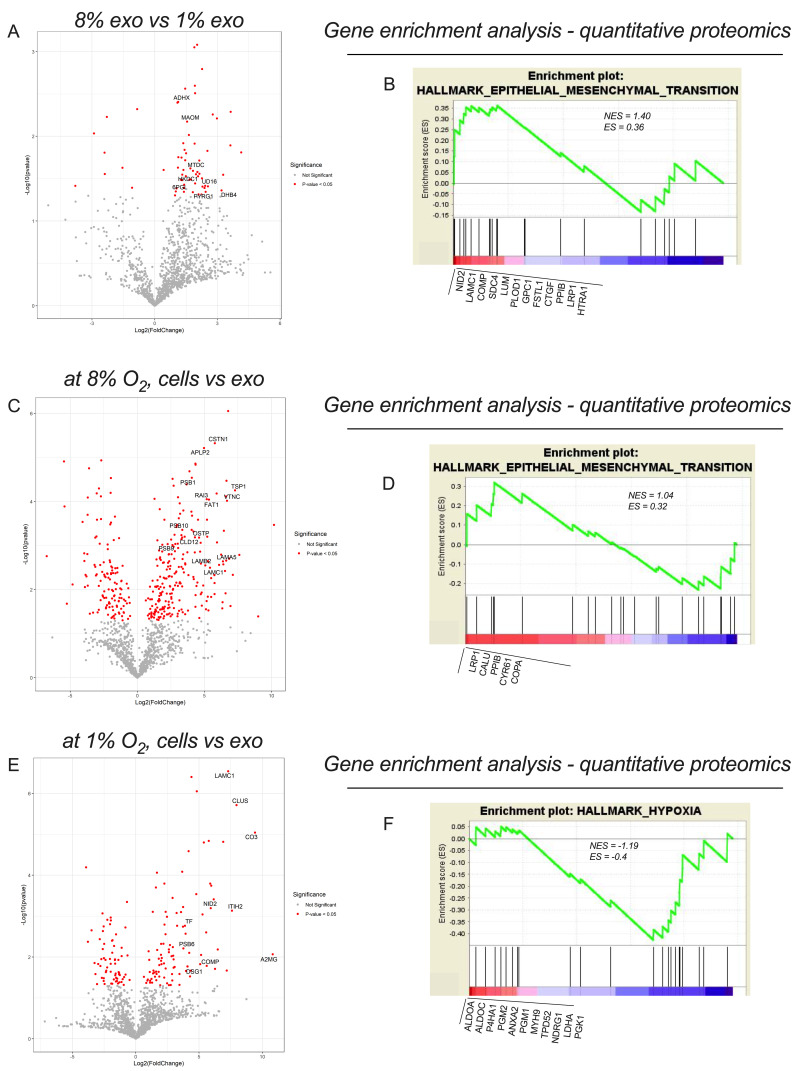
Proteomic analysis of CAOV-3 cells and their derived sEVs under 1% and 8% oxygen tension. (**A**) Volcano plot of proteins differentially expressed between sEVs secreted by the 8% O_2_ CAOV-3 cells and the 1% O_2_ CAOV-3 cells. (**B**). Volcano plot of proteins differentially expressed between the 8% O_2_ CAOV-3 cells and their derived sEVs. (**C**) Volcano plot of proteins differentially expressed between the 1% O_2_ CAOV-3 cells and their derived sEVs. (**D**–**F**) Identification of the top candidate protein using gene set enrichment analysis (GSEA) in the 1% O_2_ CAOV-3 derived sEVs compared with the 8% O_2_ CAOV-3 derived sEVs. The GSEA plots of proteomic data illustrate four gene clusters that are enriched in the 1% O_2_ CAOV-3 derived sEVs involving: (**D**) glycolysis (**E**) the epithelial–mesenchymal transition, (**F**) hypoxia. The two top networks identified using ingenuity pathway analysis are canonical pathways and diseases and biological functions analysis for 1% O_2_ sEVs in compared with 8% sEVs.

The difference in the proteomic profiles of sEVs from 1% versus 8% O_2_ is summarized in Figure 4A, as a volcano plot. A total of 65 proteins were differentially expressed in sEVs that were cultured under hypoxic conditions when compared with normoxic cells (8% O_2_) (Supplemental Table S2). Moreover, glycolysis pathway-associated proteins and detoxification enzymes were enriched in sEVs from 1% when compared with 8% O_2_ (Figure 4B and Supplemental Table S3). A specific set of proteins was selectively enriched within the sEVs when compared with their cells of origin at 8% and 1% O_2,_ indicating the specific packaging of proteins into sEVs (Figure 4C–E). 8% sEVs were enriched with proteins associated with epithelial mesenchymal transition (Figure 4D), and 1% sEVs were enriched with hypoxia association proteins (Figure 4F). Data were analyzed by GSEA and are presented in Figure 4B,D,F.

Ingenuity Pathway Analysis (IPA) was used to identify signaling pathways associated with changes in the proteomic profile in sEVs from cells cultured under hypoxic compared with normoxic conditions. The main network activated by hypoxic sEVs compared with normoxic sEVs is mitochondrial dysfunction and NRF2-mediated oxidative stress response and synaptogenesis signaling pathway (Supplemental Figure S1A). The top regulator effect networks were associated with movement of endothelial cells and invasion of cancer cells (Supplemental Figure S1B). Overall, this study suggests that when compared with sEVs from normoxic cells, sEVs from hypoxic conditions were enriched with signals associated with metastasis and glycolysis—key factors in chemoresistance [29]. This indicates that sEVs from hypoxic cells may play a role in regulating recipient-cell responses to carboplatin and induce chemoresistance in ovarian cancer.

### 2.5. Targeted Proteomic Analysis of Glycolysis in Ovarian Cancer Cells

**Figure 5:**
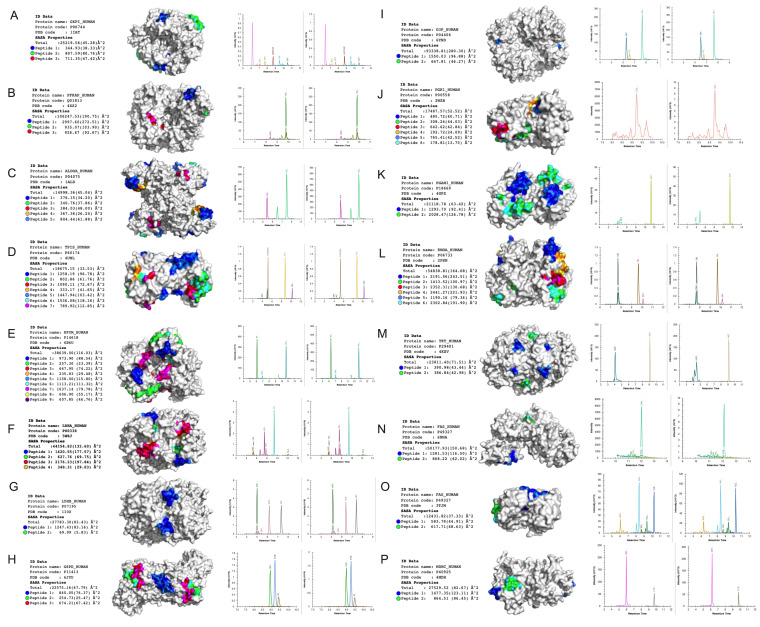
Peptides in the glycolytic enzyme structure. **Left**: The peptides are colored by the number for each glycolytic enzyme structure from the protein data bank (PDB) with the best resolution and coverture available. For each peptide, the solvent accessible surface (SASA) was calculated with the software freesasa, the average for residue is in parenthesis (**A**–**P**). The structures were prepared with pymol in surface mode and in biological assembly available from PDB (monomer, dimer, and tetramer). **Right**: MRM chromatogram of the transition used to trigger MS/MS for each protein associated with “glycolysis proteome”.

The effects of hypoxia on glycolytic pathway proteins were assessed using liquid chromatography multiple reaction monitoring (LC-MRM). Proteins involved in core glycolysis, glycolysis regulation, pentose phosphate and other metabolism pathways were selected based on previously published data [30] and are detailed in the Supplemental Table S4 and Figure 5. To ensure assay reproducibility, a Hela digest was injected at the start and in the middle of the sample set. Figure 5 show the peak areas and retention times for each of the selected peptides.

### 2.6. Effect of Hypoxia on Glycolytic Pathway Proteins in Ovarian Cancer Cell and Their Secreted Extracellular Vesicles

**Figure 6:**
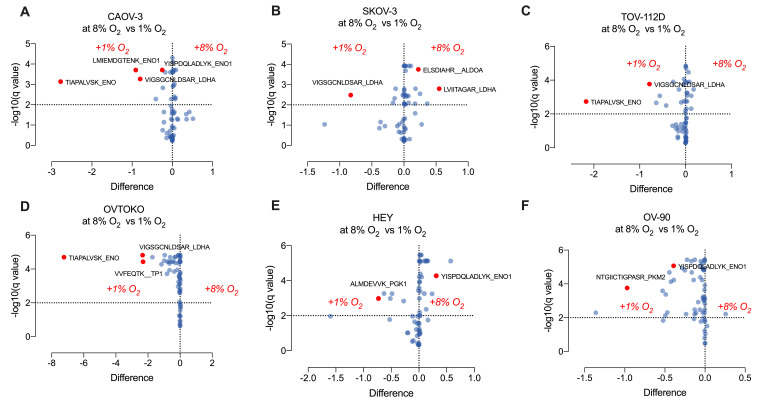
Relative quantification of the glycolysis proteome in ovarian cancer cells in response to oxygen tensions. Ovarian cancer cells were cultured under 8% or 1% oxygen, and the protein abundance associated with glycolysis were quantified using a targeting mass spectrometry approach. (**A**): CAOV-3; (**B**): SKOV-3; (**C**): TOV-112D; (**D**) OVTOKO; (**E**) HEY; and (**F**) OV-90. Volcano plots show the differences in peptides associated with proteins representatives of glycolysis proteome in cells cultured at 8% compared with cells cultured at 1% oxygen.

**Figure 7:**
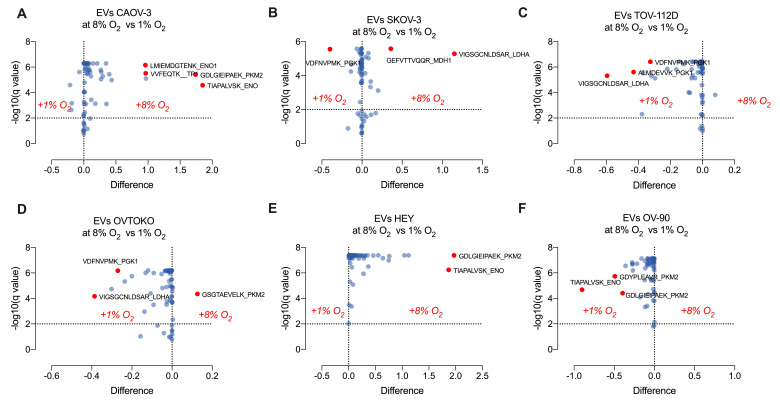
Relative quantification of the glycolysis proteome in sEVs secreted from ovarian cancer cells in response to oxygen tensions. sEVs were isolated from ovarian cancer cells cultured under 8% or 1% oxygen, and the protein abundance associated with glycolysis were quantified using a targeting mass spectrometry approach. (**A**): CAOV-3; (**B**): SKOV-3; (**C**): TOV-112D; (**D**) OVTOKO; (**E**) HEY; and (**F**) OV-90. Volcano plots show the differences in peptides associated with proteins representatives of glycolysis proteome in sEVs from cells cultured at 8% compared with cells cultured at 1% oxygen.

**Figure 8:**
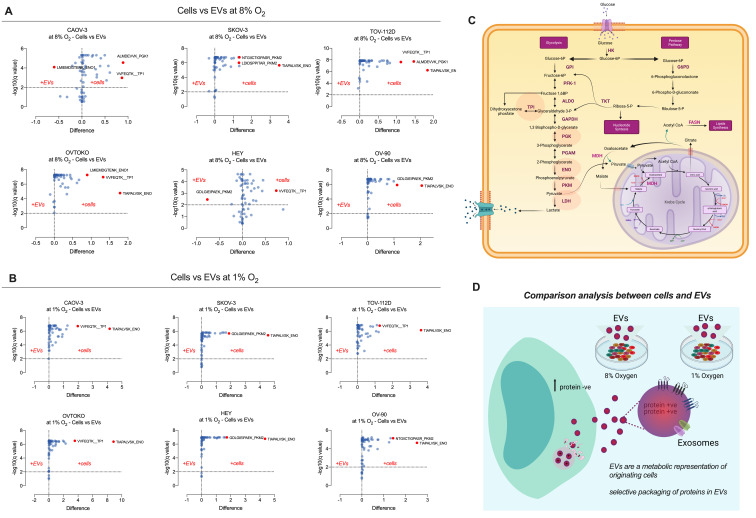
Comparison analysis of the glycolysis proteome in ovarian cancer cells versus their secreted sEVs in response to oxygen tensions. Analysis of the glycolysis proteome in ovarian cancer cells and sEVs was performed to evaluate whether changes in cells in response to normoxia or hypoxia is reflected in their secreted sEVs. (**A**) Comparison analysis of the effect of 8% oxygen in cells vs. sEVs for each cell line. (**B**) Comparison analysis of the effect of 1% oxygen in cells vs. sEVs for each cell line. (**C**) Graphical representation of proteins associated with glycolysis that changes across all the conditions studied. (**D**) Summary of the results suggesting that changes in cells are reflected in sEVS, or specific packaging of proteins in sEVs in response to oxygen tensions.

To evaluate whether low oxygen tensions (i.e., 1% oxygen) modified the glycolysis proteome in ovarian cancer cell lines, and their secreted sEVs, we performed three experimental analyses: (i) *Cellular Proteome:* The effect of hypoxia (1% O_2_ for 48 h) on the proteome of CAOV-3, SKOV-3, TOV-112D, OVTOKO, HEY and OV-90 cell lines is presented in as Figure 6A–F. Significant cell-specific differential responses (*p* < 0.05) in the expression of glycolytic pathway proteins (e.g., *ENO1, LDHA, ENO, PGK1, PKM2 TP1)* were identified; (ii) *Extracellular Vesicle Proteome:* The effect of hypoxia on extracellular vesicle-associated proteins isolated from CAOV-3, SKOV-3, TOV-112D, OVTOKO, HEY, and OV-90 cell-conditioned media are presented as volcano plot in Figure 6A–F. Specific changes associated with hypoxia for each cell line were identified and included, TPI, PGK, ENO, PKM, and LDH (*p* < 0.05); and (iii) *Comparison of Cellular and Extracellular Vesicle Proteomes:* A cell-specific comparison of the cellular and extracellular vesicle proteomes is present in Figure 7. In general, changes in the glycolysis proteome of cells are reflected in their secreted EVs under both 8%, and 1% oxygen (Figure 7A,B). Specific changes associated with each cell type were identified, in cells (e.g., TP1, and PGK1 in CAOV-3), and in EVs (e.g., PKM2 in HEY) at 8% oxygen. Specific differentially abundant proteins in cells compared with EVs were identified for each cell line (i.e., comparison analysis between cells and EVs for COV-3, SKOV-3, TOV-112D, OVTOKO, HEY, and OV-90), suggesting that proteins associated with glycolysis proteome in EVs do not reflect the cell of origin, perhaps due to the specific packaging of proteins into EVs (Figure 8C,D).

### 2.7. sEVs Released from Hypoxic Cells Confer Carboplatin Resistance to Recipient Normoxic Cells

**Figure 9:**
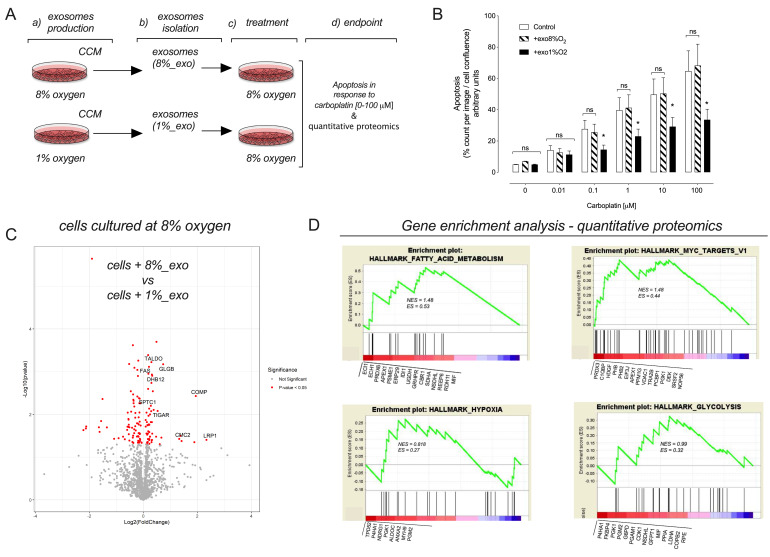
1% O_2_ sEVs induced chemoresistance in 8% O_2_ CAOV-3 cells. (**A**) Experimental strategy. (**B**) Apoptosis in 8% O_2_ CAOV-3 cells exposed to 8% O_2_ sEVs, 1% O_2_ sEVs, or a negative control (plain media). The white bar indicates cells without pre-exposure to sEVs (plain media), while the black bar indicates pre-exposure to 1% O_2_ sEVs. After 24 h, the cells were treated with different concentrations of carboplatin (0 µM, 0.001 µM, 0.01 µM, 0.1 µM, 1 µM, 10 µM, and 100 µM) in the presence of Caspase-3/7 reagent. The percentage of apoptotic CAOV-3 cells was measured using the IncuCyte™ real-time cell-imaging system every 2 h for 72 h. The data were normalized using cell confluence. The ∆ rate of cell apoptosis over time was analyzed as the area under the curve. The data are represented by the mean ± SEM (*n* = 6). (**C**) Volcano plot of proteins differentially expressed between 8% O2 CAOV-3 cells treated with 8% O_2_ sEVs and 8% O_2_ CAOV-3 cells treated with 1% O_2_ sEVs. (**D**). Identification of the top candidate proteins using gene set enrichment analysis in 8% O_2_ CAOV-3 cells treated with 1% O_2_ sEVs compared with 8% O_2_ CAOV-3 cells treated with 8% O2 sEVs. The gene set enrichment analysis plots of the proteomic data illustrate four gene clusters that are enriched in 1% O_2_ sEVs, hypoxia, glycolysis, and fatty acid metabolism. In (**B**), * *p* < 0.05 for exo1%O_2_ compared with exo8% O_2_ or control.

Next, we evaluate whether hypoxic sEVs can transfer resistance to carboplatin in target cells. *Effect of sEV on carboplatin-induced apoptosis:* The effects of hypoxia on the capacity of sEVs to inhibit carboplatin-induced apoptosis in CAOV-3 cells is presented in Figure 9. EVs isolated from CAOV-3 cells cultured under 1% significantly inhibited carboplatin-induced [0–100µM] apoptosis in normoxic CAOV-3 cells. *Effect of sEV on recipient cell proteome:* The effect of EVs isolated from CAOV-3 cells cultured under 1% on the proteome of recipient normoxic CAOV-3 cells is summarized in Supplemental Table S5 and Figure 9C. A total of 132 proteins were differentially expressed (*p* < 0.05) in cells incubated in the presence of hypoxic sEVs compared with cells incubated with normoxic sEVs. Gene enrichment analysis (GSEA) identified genes related to oxygen-monitoring mechanisms, including hypoxia-inducible factors (HIFs), the major components of hypoxia signaling pathways, glycolysis, fatty acid synthesis, and protein secretion (Figure 9D). The data obtained are consistent with the hypothesis that oxygen tension regulates the packing of specific proteins into sEVs that are involved in the metabolic adaptation of cancer to enhance glycolytic pathways and that they play a role in inducing carboplatin resistance in recipient cells.

### 2.8. Analysis of Circulating sEVs in Patients with Ovarian Cancer

**Figure 10:**
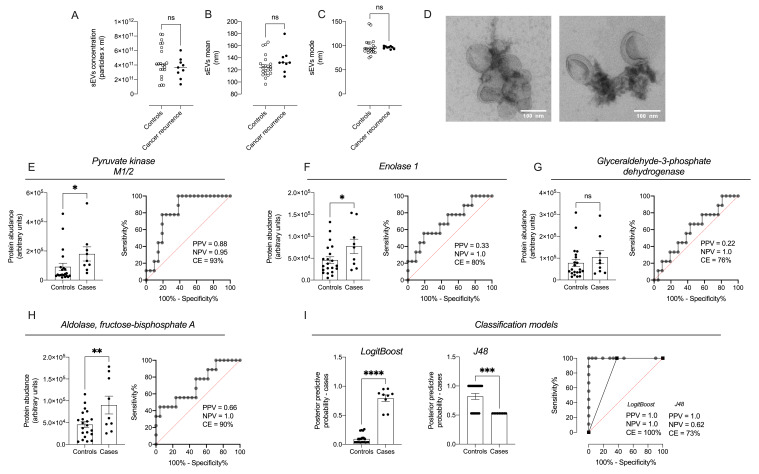
Receiver operating characteristic (ROC) curves illustrating the ability of proteins within sEVs to distinguish patients with cancer recurrence. sEVs were isolated from plasma obtained from patients with cancer recurrence and control. sEVs were characterised uisng nanoparticle tracking analysis by (**A**) concentration; (**B**) mean; (**C**) mode; and (**D**) electron microscopy. The levels of selected proteins, (**E**) pyruvate kinase M1/2; (**F**) enolase 1; (**G**) glyceraldehyde-3-phosphate dehydrogenase; and (**H**) aldolase, fructose-bisphosphate were quantified by SWATH analysis. In (**E**–**H**), left: levels of pyruvate kinase M1/2, enolase 1, glyceraldehyde-3-phosphate dehydrogenase, or aldolase, fructose-bisphosphate (**A**); and right: ROC curve for each protein. (**I**) Classification model based on the quantification of all proteins in sEVs and developed using LogitBoost regression analysis, or J48 decision tree model. PPV = positive predicted values; NPV = Negative Predictive Values; CE: classification Efficiency. * *p* < 0.05, ** *p* < 0.005, *** *p* < 0.0005 and **** *p* < 0.0001 for cases vs. controls. Ns = non-significant.

To determine if changes in protein expression observed in vitro experiments are recapitulated in vivo, sEVs were isolated from the plasma of patients with ovarian cancer recurrence (*n* = 9), and controls (i.e., without ovarian cancer, *n* = 22) after 12 months from initial diagnosis (Figure 10) (Table S6). No differences were identified in the concentration, mean, and mode of the circulating sEVs from patients with cancer recurrence and controls (Figure 10A–C). Morphological analysis using electron microscopy showed vesicles of around 100 nm (Figure 10D). Information-Dependent acquisition (IDA) mass spectrometry analysis was performed and identified 353 total proteins (Supplemental Table S7). Interestingly, of the 353 proteins identified in sEVs from plasma samples, a total 89 proteins are present in sEVs from CAOV-3 cells cultured at 1% oxygen (Supplemental Figure S2). An Ingenuity Pathway Analysis library identified 4 exosome-associated glycolysis pathway proteins: pyruvate kinase M1/2, enolase 1, glyceraldehyde-3-phosphate dehydrogenase, and aldolase fructose-bisphosphate (Supplemental Figure S3). The relative abundance of these proteins in patients with ovarian cancer recurrence and controls was established by comparison using SWATH and expression profiling against the IDA library (Figure 10E–H). The expression of pyruvate kinase M1/2, enolase 1, and aldolase fructose-bisphosphate were significantly higher in circulating sEVs of patients with ovarian cancer recurrence compared to the controls (Figure 10E–I). No difference in the expression of glyceraldehyde-3-phosphate dehydrogenase was identified (Figure 10G).

The classification efficiency (i.e., the proportion of ovarian cancer recurrence cases correctly identified) by measuring pyruvate kinase M1/2, enolase 1, glyceraldehyde-3-phosphate dehydrogenase, and aldolase fructose-bisphosphate A, individually, and in combination, was assessed by ROC curve analysis (Figure 10E–I). The area under the ROC curves (AUC) for pyruvate kinase M1/2, enolase 1, glyceraldehyde-3-phosphate dehydrogenase, and aldolase, fructose-bisphosphate A, were 0.80 ± 0.08 (AUC ± SD), 0.69 ± 0.10, 0.60 ± 0.10, 0.71 ± 0.10, respectively. The classification efficiencies for pyruvate kinase M1/2, enolase 1, glyceraldehyde-3-phosphate dehydrogenase, and aldolase fructose-bisphosphate A were 93%, 80%, 76%, and 90%, respectively (Figure 10E–H, right). Multivariate models (LogitBoost and J48 decision tree) were developed using WEKA software. The AUCs were 1.0 and 0.80 (Figure 10I) and the overall classification accuracy was 100% and 73% for the LogitBoost and J48 models, respectively. The data suggest that the analysis of proteins associated with glycolysis encapsulated in circulating sEVs might be used as potential biomarkers to identify women at risk of cancer recurrence and may be useful to determine treatment response in ovarian cancer.

## 3. Discussion

The development of chemoresistance in patients with cancer is a major factor limiting survival [31]. Elucidating the fundamental mechanisms by which cancer cells acquire resistance to chemotherapeutic agents, therefore, is requisite to developing more efficacious treatments. Hypoxia is a common feature of malignant tumors [32] and is associated with reprogramming of cellular metabolism to favor glycolytic energy production, increased invasiveness, metastatic potential, angiogenesis, and reduced apoptosis and chemoresistance [32,33,34,35]. Interestingly, small extracellular vesicles like exosomes have been associated with platinium resistance in ovarian cancer [36]. This study aimed to test the hypothesis that hypoxia-induced chemoresistance is propagated within ovarian tumors by extracellular vesicle signaling between cancer cells. In this study, we used the terminology sEVs as recommended by the International Society of Extracellular Vesicles, due to the mixed origin of EV preparations and the lack of specific EV subtype markers [37].

The data obtained using a panel of nine epithelial ovarian cancer cell lines, confirm that carboplatin resistance (as defined by cell the IC_50_ for cell survival) is regulated by oxygen tension but varies up to 3-fold between cell lines (IC_50_ = 8.7 µM for OVCA-420 to IC_50_ = 28.5 µM for CAOV-3). The incubation of cells under low oxygen tension induces the cellular expression of a cassette of proteins associated with hypoxia (e.g., HIF-1), epithelial mesenchymal transition (e.g., GPC1, PPIB), glycolysis (e.g., CPC1, FKBP4) and MYC targets (e.g., XP01, and G3BP1), and increases the release of extracellular vesicles by 2-fold (as indicated by the concentration of extracellular vesicles in cell-conditioned media). Hypoxia-induced cellular proteins were enriched in the extracellular vesicles released from these cells. The treatment of carboplatin-sensitive ovarian cancer cells with extracellular vesicles isolated from cells incubated under low oxygen tension conferred carboplatin resistance. These data support the hypothesis that ovarian cancer cells utilize extracellular vesicle signaling between cancer cells to propagate chemoresistance. Furthermore, the data obtained are consistent with recent studies that implicate extracellular vesicles in hypoxia-induced metabolic changes within the pre-metastatic milieu [38,39,40] and in the acquisition of chemoresistance [41,42,43,44,45,46].

While the mechanisms by which extracellular vesicles derived from hypoxic ovarian cancer cells induce chemoresistance in recipient cells remains to be elucidated, the protein cargo they carry has been implicated in metabolic and oncogenic pathways. For example, HIF-1, increases the flux of the glycolysis pathway, which in turn decreases the flux of the tricarboxylic acid cycle (TCA). Decreased TCA flux reduces mitochondrial reactive oxygen species (ROS) production but maintains matrix ATP concentrations under energy-limited conditions [47]. HIF-1 also increases the flux of the serine synthesis pathway, inducing the production of antioxidant products, such as NADPH and glutathione, to neutralize ROS [47]. In this study, 3-hydroxyacyl-CoA dehydrogenase (HAD) was significantly upregulated. HAD belongs to a family of oxidoreductases that catalyzes 3-hydroxy-2-methylbutanoyl-CoA in the third step of beta-oxidation and produces NADPH and 2-methyl-3-COA.

Similarly, hexokinase (HK), UDP-glucuronosyltransferase 1–6 (UD16), 6-phosphogluconolactonase (6PGL), and CTP synthase 1 (PYRG1), the key enzymes involved in the glycolysis and biosynthesis of phospholipids and nucleic acids [48], were significantly enriched in the sEVs from hypoxic cells. Patra et al. demonstrated that HK positively correlates with cancer cell survival through the initiation of glycolysis, an effect that is abolished when HK is knocked out using an in vivo model [49]. HK expression is a key factor in inducing tumor progression whilst depletion of HK assists in restoring the balance between glycolysis and OXPHOS pathways, leading to the upregulation of mitochondrial biogenesis and induction of intrinsic apoptotic pathways [50]. HK is also involved in chemoresistance via the enhancement of the autophagy of cisplatin in ovarian cancer [51]. The enrichment of proteins that are involved in NADH/NADPH productions (i.e., alcohol dehydrogenase class-3 (ADH5), NAD-dependent malic enzyme (MAMO), bifunctional methylenetetrahydrofolate dehydrogenase/cyclohydrolase (MTDC) and peroxisomal multifunctional enzyme type 2 (DHB4)) was also detected in hypoxic sEVs. This enrichment could potentially act as a protective mechanism for the targeted cells as it has been shown that these enzymes reduce oxidative stress under low oxygen consumption. We, therefore, suggest that the presence of oncogenic molecules secreted from hypoxic cells via sEVs initiate the metabolic changes of normoxic cells, upregulate the glycolysis pathway, and drive physiological responses such as cell survival and chemoresistance.

In addition to proteins, EVs can carry a diverse range of molecules, such as lipids or microRNAs that can mediate chemoresistance by interacting with the immune system [52]. Cytotoxic chemotherapy induces dynamic changes in the cellular immune landscape from within the tumor microenvironment, impacting overall survival outcomes [53,54]. EVs obtained from ovarian tumors were found to be immunosuppressive [55], causally linked by their cargoes of lipid phosphatidylserine (PS) [56,57] and ganglioside GD3 [58,59]. Furthermore, the microRNA-940 was found to be highly expressed in EVs derived from ovarian cancer cells under hypoxic conditions [60], inducing macrophages to remodel a pro-tumorigenic microenvironment. Future analyses investigating the role of EVs from hypoxic ovarian cancer cells could also characterize non-protein cargoes such as PS, ganglioside GD3 or microRNAs. Thus, further studies are required to determine the role of other molecules within sEVs from hypoxic conditions on conferring resistance to carboplatin in target cells.

The data obtained define an extracellular vesicle chemoresistance protein profile that may be of clinical utility as an aid in the management of patients with disease recurrence. More than 60% of ovarian cancer patients that present with advanced stage disease develop recurrent chemoresistant disease [61]. In this study, we identified an increased expression of pyruvate kinase M1/2, enolase 1, and aldolase, fructose-bisphosphate A, in circulating extracellular vesicles of patients with ovarian cancer recurrence compared to the controls. Previously, these enzymes have been implicated in metabolic adaption, tumorigenesis, chemoresistance and patient survival [62,63,64,65,66,67,68,69,70,71,72,73,74,75,76,77,78]. Multivariate models based on the expression of these proteins in extracellular vesicle delivered classification efficiencies of 73–100%. A larger patient study is warranted to validate these findings clinically.

In conclusion, this study advances our understating of the involvement of extracellular vesicle signaling pathways in the transfer of chemoresistance between ovarian cancer cells. Such pathways may be targeted for therapeutic intervention and/or improved patient management.

## 4. Material and Methods

### 4.1. Cell Culture

In this study, human ovarian cancer cell lines: TOV-112D, OVCAR-3, OV90, HEY, OVCA-429, OVTOKO, SKOV-3, and OVCA-420 were used. All cell lines, except OVCA-429, were maintained in RPMI media supplemented with 10% heat-inactivated fetal bovine serum (FBS) (PAA Laboratories Pty Ltd., Morningside, QLD, Australia), 1000 U/mL antibiotic-antimycotic, (Gibco, Life Technologies, Carlsbad, CA, USA). OVCA-429 was maintained in DMEM media (Life Technologies, Carlsbad, CA, USA) with 10% heat-inactivated FBS (PAA Laboratories Pty Ltd., Morningside, QLD, Australia), and 1000 U/mL antibiotic-antimycotic solution (Gibco, Life Technologies, Carlsbad, CA, USA). Cells were cultured as an T175 flasks adherent monolayer under 8% O_2_ to simulate normoxic, and under 1% O_2_ in a humidified chamber to simulate hypoxia.

### 4.2. Apoptosis Assay

A total of 3 × 10^4^ of TOV-112D, OVCAR-3, OV90, HEY, OVCA-429, OVTOKO, SKOV-3 and OVCA-420, cells were plated in 96-well plates (Corning Life Science, Tewksbury, MA, USA) under 8% O_2_ (normoxic) or 1% O_2_ (hypoxia). After 24 h, cells were treated with different concentrations of carboplatin (0.01–100 μM) for 72 h. The effect of carboplatin on cell apoptosis was determined by quantifying Caspase3/7 (ThermoFisher, Waltham, MA, USA) using the real-time cell imaging system, IncuCyte™ (Essen BioScience, Ann Arbor, MI, USA). Images were captured every two hours to monitor apoptosis.

### 4.3. sEVs Isolation and Characterization

Cells grown to 70% confluence in T175 flasks were washed twice with phosphate-buffered saline (PBS) before FBS-free media was added. The cells were then incubated for 48h before conditioned media was collected for sEV isolation. sEV were isolated by differential centrifugation and ultrafiltration as previously published [16]. Briefly, cell conditioned media or plasma were centrifuged at 800× *g* for 10 min, 2000× *g* for 10 min at 4 °C, and 12,000× *g* for 10 min at 4 °C to remove cells and debris. The resultant supernatant was centrifuged at 100,000× *g* for 2 h at 4 °C with the Type 70.1 Ti fixed angle rotor (Beckman Coulter, CA, USA) to pellet the sEVs. The resultant pellet was resuspended in 10 mL PBS, filtered through a 100 kDa Amicon^®^ Ultra-15 Centrifugal Filter Units (MERCK, Bayswater, VIC, Australia), and centrifuged at 4000× *g* for 30 min. The sEVs were collected and stored at −80 °C for further experiments. sEVs were characterized according to the recommendations of the International Society of Extracellular Vesicles, by size distribution, abundance of proteins (i.e., CD63 and TSG101) associated with sEVs, and by a morphological assessment involving Nanoparticle Tracking Evaluation (NTA), Western blot, and electron microscopy [37].

### 4.4. Sample Preparation for SWATH) Analysis

#### 4.4.1. Protein Extraction for Tandem Mass Spectrometry (MS/MS)

Protein was extracted from the following sources by SWATH as described previously [16]: ovarian cancer cells cultured under either 1% O_2_ (hypoxia) or 8% O_2_ (normoxic); sEVs derived from normoxic and hypoxic cells; and CAOV-3 cells cultured under 8% O_2_ and treated with either 1% O_2_ sEVs (1% O_2_ sEVs + 8% O_2_ cells) or 8% O_2_ sEVs (8% O_2_ sEVs + 8% O_2_ cells). Briefly, cells were washed twice with PBS (Sigma Aldrich) and lysed using Radio-Immunoprecipitation Assay (RIPA) buffer (Sigma Aldrich). The cell lysate was transferred to a pre-chilled microcentrifuge tube and centrifuged at 15,000× *g* for 15 min at 4 °C. The supernatant containing total cellular proteins was collected and quantified using the DC™ Protein Assay kit (BIO-RAD, Gladesville, NSW, Australia). Exosome fractions were lysed using 8M urea in 50 mM triethylammonium bicarbonate at pH 8.0.

#### 4.4.2. Ion Library Generation and SWATH

Protein (20 µg) from individual samples was reduced, alkylated, and trypsinized in triplicate using the filter-aided sample preparation (FASP) method in order to generate an ion library for SWATH mass spectrometry (MS) analysis. The resulting peptide samples were combined and processed in an information-dependent acquisition (IDA) on an AB Sciex 5600 TripleTOF MS with the top 20 precursor ions automatically selected for fragmentation and processed. For SWATH analysis, a global false discovery rate (FDR) of 1% was used as the threshold for the number of proteins for import to ensure the highest quality ion library. SWATH acquisition was as described by Menon et al., [79], operating in a looped product ion mode. Using an isolation width of 26 Da (25 Da of optimal ion transmission efficiency and 1 Da for the window overlap), a set of 32 overlapping windows (1 *m*/*z* overlap) was constructed covering the mass range 400 to 1200 *m*/*z*.

#### 4.4.3. Data Processing

To generate the protein library, ProteinPilot (version 4.5b) software and the Paragon™ Algorithm were used to search against a human SwissProt database. For SWATH processing, the SWATH Acquisition Microapp (version 2.0) within PeakView (version 2.2) was used. An FDR threshold of 1% was applied by setting three peptides per protein with the retention time manually realigned with a minimum of five peptides with consistently high signal intensities and distributed along the time axis. The resulting peak area for each protein after SWATH processing was exported to MakerView version 1.3.1 (SCIEX) for statistical analysis. The resulting data was normalized using Total Area Sum (TAS). The coefficient of variation in the peptide abundance across the samples was established by comparing the SWATH peptide ion against the IDA library. Finally, unpaired Student’s t-tests were performed with *p* < 0.05 considered statistically significant.

#### 4.4.4. Target Peptide Set Selection for “Glycolysis Proteome”

The proteins involved in core glycolysis, glycolysis regulation, pentose phosphate, and other metabolism pathways were selected based on a previous study [30]. Also included were five commonly used reference proteins. The FASTA protein sequence for all proteins were imported into Skyline software (version 20.2.1.404) for method development and data processing. For selecting the optimal fragment ions, a targeted MRMHR experiment was performed using the pre-selected peptides and their corresponding masses as per the above study. A commercially available Hela tryptic digest (500 ng) was injected two times into a TripleTOF 5600 mass spectrometer (AbSciex) connected to a NanoLC 400 system with an analytical MicroLC column ChromXP C18CL, 120A, 150 × 0.3 mm (Eksigent). Chromatography was performed in a trap-elution mode with solvent A (100% H_2_O, 0.1% FA) and solvent B (100% ACN, 0.1% FA). Peptide separation was performed at a total flow rate of 5 µL/mL according to the gradient conditions listed the Supplemental Table S8. Afterwards, a full scan data extraction from the raw. wiff files were conducted in Skyline. The fragment ions were manually inspected, and the presence of consecutive high mass y ion series gives evidence of the correct identification of the peptide. The top three fragment ions were elected for the MRM experiment based on the highest co-eluting y ions’ intensities.

The MRM experiments were performed on a 5500 QTRAP hybrid triple quadrupole/linear ion trap mass spectrometer (ABSciex), equipped with an Eksigent MicroLC 200 System. Hela digest was again injected, with the retention times of the peptides used to build a scheduled method with a two-minute window. Cells or cell derived EVs were digested using FASP as previously described by our group [80]. Tryptic digests of cells or exosomes were resuspended in 20 uL of H_2_O, 0.1% FA of which 5 uL was injected and separated by RP-HPLC in an analytical MicroLC column HALO™ C18 2.7 µm, 90A 0.5 × 100 mm (Eksigent). Chromatography was performed with solvent A (100% H_2_O, 0.1% FA) and solvent B (100% ACN, 0.1% FA) at a total flow rate of 15 µL/mL according to the gradient conditions listed in Supplemental Table S9. To ensure the assay’s reproducibility, a commercially available hela peptide digest (ThermoFisher) was injected at the start and in the middle of the sample set (Figure 5). The peak areas and retention times for each of the selected peptides.

### 4.5. Gene Set Enrichment Analysis (GESA)

To determine the genes associated with chemoresistance and cancer progression in 1% O_2_ CAOV-3 cells, 8% O_2_ CAOV-3 cells, 1% O_2_ sEVs, 8% O_2_ sEVs, 1% O_2_ sEVs + 8% O_2_ CAOV-3 cells, and 8% O_2_ sEVs + 8% O_2_ CAOV-3 cells, Gene Set Enrichment Analysis (GSEA, version 3.0) was performed [81] using the normalized SWATH results of these samples. The protein expression data was processed using the hallmark gene sets within the MSigDB database v6.2 with permutations set at 1000 and a Signal2Noise metric for ranking genes. Default values were selected for all other parameters.

### 4.6. Effect of sEVs on the Response of CAOV-3 Cells to Carboplatin

CAOV-3 cells (3 × 10^4^) were plated in 96-well plates (Corning Life Science, Tewksbury, MA, USA) and cultured under either 8% or 1% O_2_. After 24 h, cells cultured under 8% O_2_ were co-incubated in the absence or presence of sEVs from hypoxic (1% O_2_) CAOV-3 cells for 24 h. The cells were then treated with different concentrations of carboplatin (10–100 μM) for 48 h. The effect of carboplatin on cell apoptosis was determined by quantifying Caspase3/7 using the real-time cell imaging system, IncuCyte™ (Essen BioScience, Ann Arbor, MI, USA). Images were captured every two hours to monitor apoptosis.

### 4.7. Patient Derived Specimens

Using an Oregon Health & Science University (OHSU) Institutional Review Board approved protocol (OHSU #0921), the participating women consented for blood draw (10mls in sodium-citrate tube) before surgery for suspected ovarian adenocarcinoma. This study was approved by the Human Research Ethics Committee of the the University of Queensland (2021/HE000319). Platelet poor plasma was made by centrifugation at 2500× *g* for 10 min and 0.5 mL aliquots were banked at −80 °C. These banked coded plasma samples were provided by OHSU for experimental analysis, linked to a coded database without patient health identifiers extracted from the electronic medical record. Study subject coded data included patient age, pathological diagnosis (e.g., benign cystadenoma, borderline tumor, primary ovarian carcinoma including differentiation and grade, or metastatic carcinoma including classification) that was confirmed by a board certified anatomic pathologist with expertise in gynecologic pathology (Dr. Morgan, OHSU) [82], clinical stage, whether the patient was optimally debulked (yes or no), interval from blood collection to follow up assessment, and clinical outcome at follow up (recurrent disease (RD), dead of disease (DOD), and alive without disease (AWOD)).

### 4.8. Statistical Analysis

Data are represented as mean ± SEM. Comparisons between two groups were performed by unpaired Student’s *t*-tests. Multiple groups were compared using the analysis of variance (ANOVA). Post hoc analyses were used for pairwise comparisons (Bonferroni correction test). Statistical significance was defined as at least *p* < 0.05. Statistical analyses were preformed using commercially available programs Stata 11 (StatCorp, College Station, TX, USA) and Prism 6 (GraphPad Inc, La Jolla, CA, USA).

## 5. Conclusions

Hypoxia induces changes in the composition and bioactivity of small EVs like exosomes, that confer carboplatin resistance in recipient target cells. These findings provide a new insight into tumor cell EV signaling and identify a putative theranostic target for the prediction and prevention of disease recurrence.

## Data Availability

All data generated during this study are available within the article and its supporting information. Further details are available from the corresponding author on reasonable request.

## Supplementary Materials

The following are available online at https://www.mdpi.com/article/10.3390/cancers13143388/s1, Figure S1: Ingenuity Pathway Analysis (IPA) was used to identify signaling pathways associated with changes in proteomic profile in sEVs from cells cultured under hypoxic compared with normoxic conditions. (A) Canonical Pathways, and (B) Diseases and Bio Functions. Figure S2. Analysis of the protein identified in small extracellular vesicles isolated from cells and plasma. sEVs were isolated from CAOV-3 cells cultured at 1% oxygen, and plasma obtained from patients with ovarian cancer, and global proteomic identification was performed. Venn diagram shown the unique and common protein identified in sEVs from plasma, and from cell-conditioned media. Figure S3. An Ingenuity Pathway Analysis library in circulating small extracellular vesicles identified 4 sEV-associated glycolysis pathway proteins: pyruvate kinase M1/2, enolase 1, glyceraldehyde-3-phosphate dehydrogenase, and aldolase fructose-bisphosphate. Figure S4. Uncropped Western blot images. Table S1. Differentially expressed proteins in 1% oxygen CAOV-3 cells compared to 8% oxygen CAOV-3 cells. Table S2: Differential expressed proteins in sEVs derived from 1% CAOV-3 cells in compared to exosomes derived from 8% CAOV-3 cells. Table S3. A comparison of proteins differentially expressed in 1% O2 sEVs and 8% O2 sEVs. Table S4. Target Set of Metabolic and Reference Proteins. Table S5. Differentially expressed proteins in cells incubated with hypoxic exosomes compared to cells incubated with normoxic sEVs. Table S6. Clinical characteristics of the patients involved in this study. Table S7. Proteins identified in circulating sEVs isolated from plasma. Table S8. Peptide separation was performed at a total flow rate of 5 μL/mL according to the gradient conditions listed below. Table S9. Chromatography was performed with solvent A (100% H_2_O, 0.1% FA) and solvent B (100% ACN, 0.1% FA) at a total flow rate of 15 μL/mL according to the gradient conditions listed below.
