# Supplementary material for: Extracellular Vesicle Transmission of Chemoresistance to Ovarian Cancer Cells Is Associated with Hypoxia-Induced Expression of Glycolytic Pathway Proteins, and Prediction of Epithelial Ovarian Cancer Disease Recurrence
Source: Cancers (Basel). 2021 Jul 6;13(14):3388. doi: 10.3390/cancers13143388 (PMC8305505; doi:10.3390/cancers13143388)
Supplement: Supplementary file 1 [file cancers-13-03388-s001.zip › cancers-1278965-supplementary.pdf]

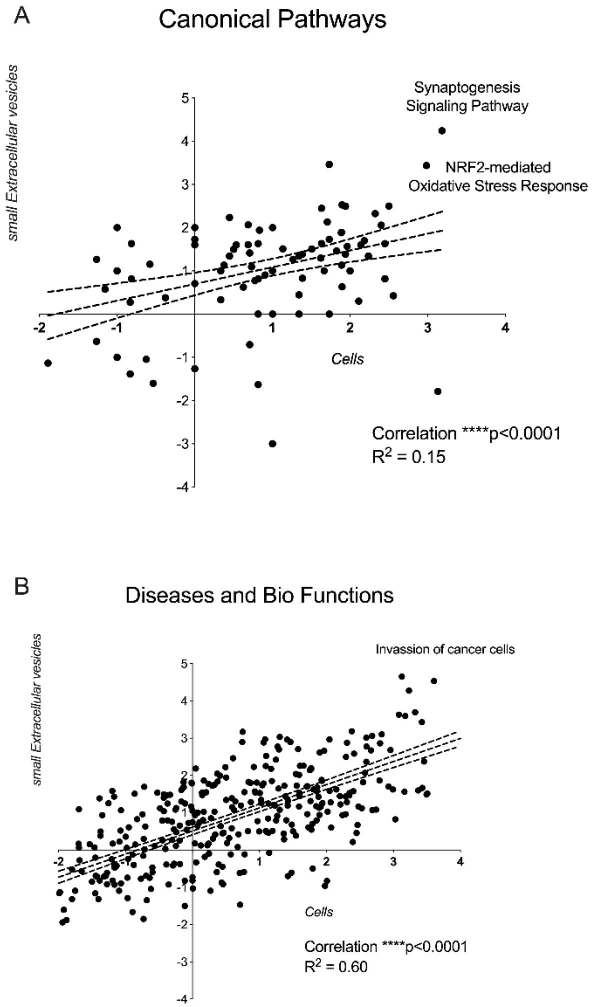

Supplemental Figure S1. Ingenuity Pathway Analysis (IPA) was used to identify signaling pathways associated with changes in proteomic profile in sEVs from cells cultured under hypoxic compared with normoxic conditions. A) Canonical Pathways, and B) Diseases and Bio Functions.

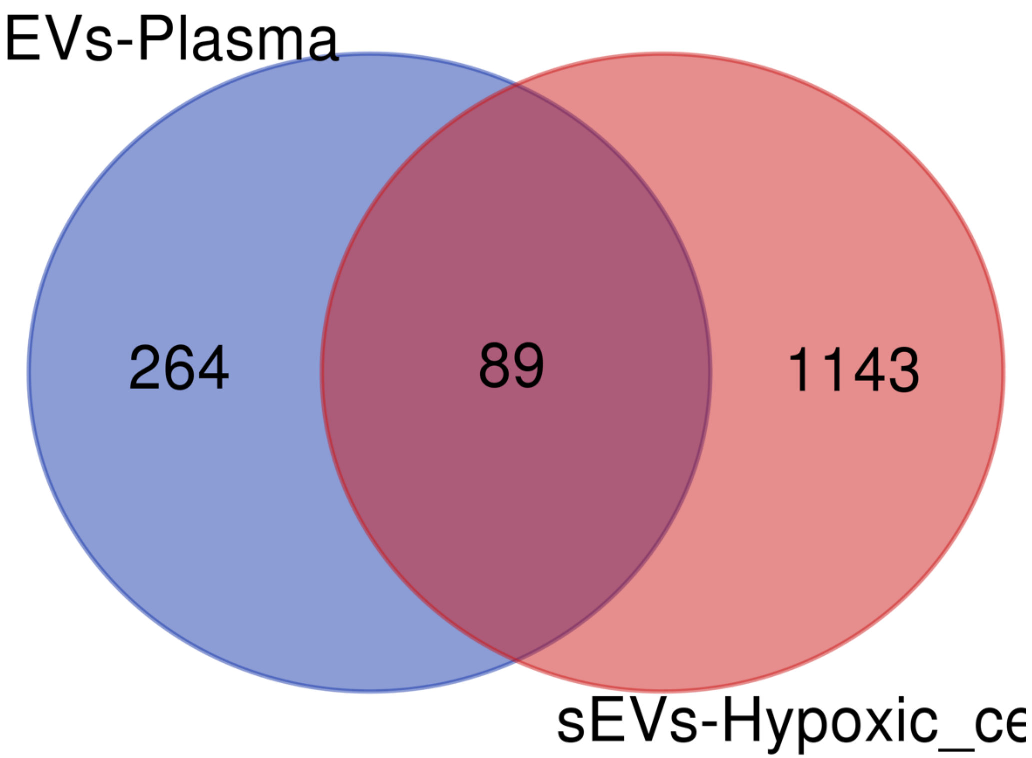

Supplemental Figure S2. Analysis of the protein identified in small extracellular vesicles isolated from cells and plasma. sEVs were isolated from CAOV-3 cells cultured at 1% oxygen, and plasma obtained from patients with ovarian cancer, and global proteomic identification was performed. Venn diagram shown the unique and common protein identified in sEVs from plasma, and from cell-conditioned media.

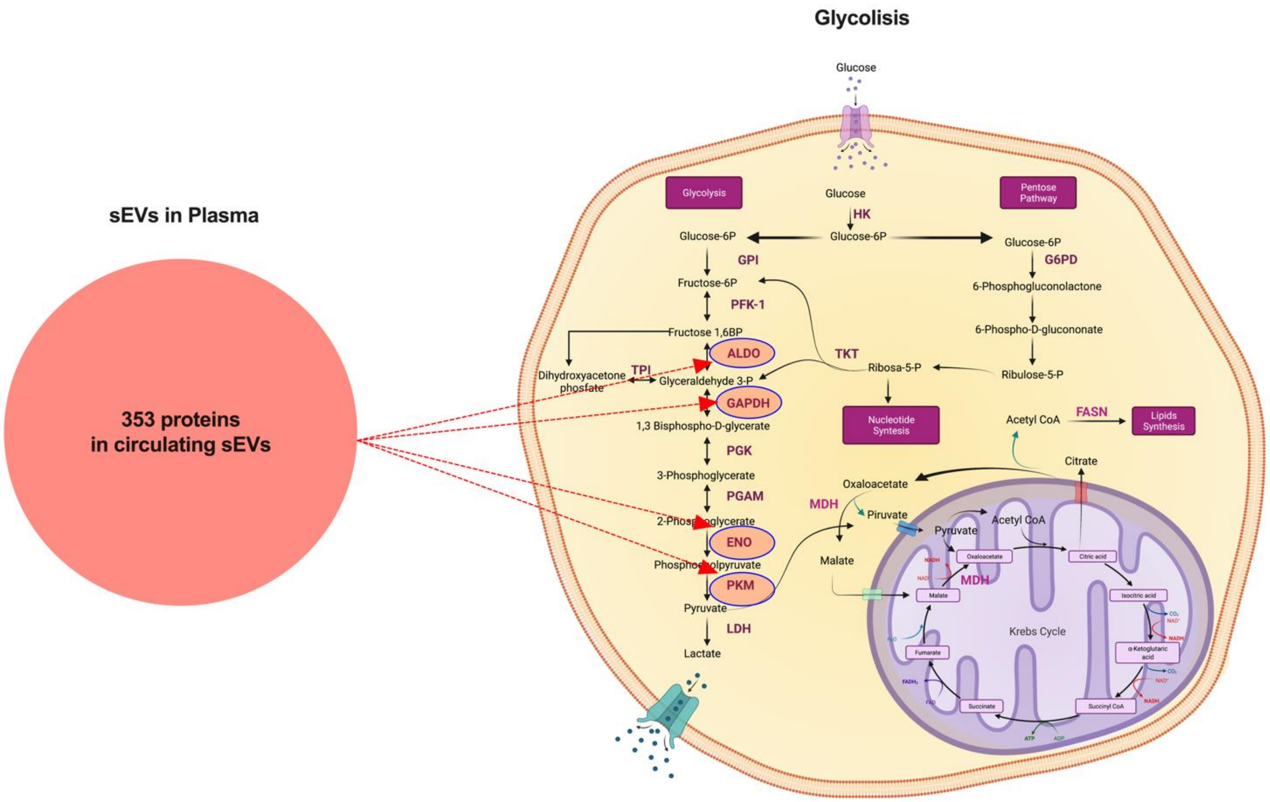

Supplemental Figure S3. An Ingenuity Pathway Analysis library in circulating small extracellular vesicles identified 4 sEV-associated glycolysis pathway proteins: pyruvate kinase M1/2, enolase 1, glyceraldehyde-3-phosphate dehydrogenase, and aldolase fructose-bisphosphate.

Table S1. Differentially expressed proteins in 1% oxygen CAOV-3 cells compared to 8% oxygen CAOV-3 cells.

Mapped IDs	Peak Name	Gene Name/ Gene Symbol/ Ortholog	PANTHER Family /Subfamily	Pather Protein Class	p-value	Fold Change
**P50402**	EMD_HUMAN	Emerin;EMD;ortholog	EMERIN (PTHR15171:SF2)		0.0007	0.079345782
**Q9P2R7**	SUCB1_HUMAN	Succinate--CoA ligase [ADP-forming] subunit beta, mitochondrial;SUCLA2;ortholog	SUCCINATE--COA LIGASE [ADP-FORMING] SUBUNIT BETA, MITOCHONDRIAL (PTHR11815:SF14)	ligase(PC00142)	0.00216	4.721641365
**P49588**	SYAC_HUMAN	Alanine--tRNA ligase, cytoplasmic;AARS1;ortholog	ALANINE--TRNA LIGASE, CYTOPLASMIC (PTHR11777:SF36)	aminoacyl-tRNA synthetase(PC00047)	0.00603	1.305535801
**Q9Y678**	COPG1_HUMAN	Coatomer subunit gamma-1;COPG1;ortholog	COATOMER SUBUNIT GAMMA-1 (PTHR10261:SF3)	vesicle coat protein(PC00235)	0.01021	0.589200077
**Q9Y376**	CAB39_HUMAN	Calcium-binding protein 39;CAB39;ortholog	CALCIUM-BINDING PROTEIN 39 (PTHR10182:SF11)		0.01282	5.893735567
**Q08257**	QOR_HUMAN	Quinone oxidoreductase;CRYZ;ortholog	QUINONE OXIDOREDUCTASE (PTHR44154:SF1)	oxidoreductase(PC00176)	0.01318	0.654118587
**Q9BRP8**	PYM1_HUMAN	Partner of Y14 and mago;PYM1;ortholog	PARTNER OF Y14 AND MAGO (PTHR22959:SF0)		0.01539	0.488257721
**P11279**	LAMP1_HUMAN	Lysosome-associated membrane glycoprotein 1;LAMP1;ortholog	LYSOSOME-ASSOCIATED MEMBRANE GLYCOPROTEIN 1 (PTHR11506:SF27)	membrane trafficking regulatory protein(PC00151)	0.01543	0.10021624
**P22626**	ROA2_HUMAN	Heterogeneous nuclear ribonucleoproteins A2/B1;HNRNPA2B1;ortholog	HETEROGENEOUS NUCLEAR RIBONUCLEOPROTEINS A2/B1 (PTHR48026:SF13)		0.01563	0.22965286
**P52823**	STC1_HUMAN	Stanniocalcin-1;STC1;ortholog	STANNIOCALCIN-1 (PTHR11245:SF1)	peptide hormone(PC00179)	0.01617	2.894444401
**Q8NBS9**	TXND5_HUMAN	Thioredoxin domain-containing protein 5;TXNDC5;ortholog	THIOREDOXIN DOMAIN-CONTAINING PROTEIN 5 (PTHR45672:SF3)	chaperone(PC00072)	0.01763	0.805404588
**P17936**	IBP3_HUMAN	Insulin-like growth factor-binding protein 3;IGFBP3;ortholog	INSULIN-LIKE GROWTH FACTOR-BINDING PROTEIN 3 (PTHR11551:SF3)	protease inhibitor(PC00191)	0.01797	2.789064867
**Q86TG7**	PEG10_HUMAN	Retrotransposon-derived protein PEG10;PEG10;ortholog	RETROTRANSPOSON-DERIVED PROTEIN PEG10 (PTHR15503:SF11)		0.01974	0.143728947
**P26006**	ITA3_HUMAN	Integrin alpha-3;ITGA3;ortholog	INTEGRIN ALPHA-3 (PTHR23220:SF89)	integrin(PC00126)	0.02445	0.411870465
**O94925**	GLSK_HUMAN	Glutaminase kidney isoform, mitochondrial;GLS;ortholog	GLUTAMINASE KIDNEY ISOFORM, MITOCHONDRIAL (PTHR12544:SF49)	hydrolase(PC00121)	0.02659	0.664538378
**P36542**	ATPG_HUMAN	ATP synthase subunit gamma, mitochondrial;ATP5F1C;ortholog	ATP SYNTHASE SUBUNIT GAMMA, MITOCHONDRIAL (PTHR11693:SF31)	ATP synthase(PC00002)	0.02669	2.012627322
**Q99961**	SH3G1_HUMAN	Endophilin-A2;SH3GL1;ortholog	ENDOPHILIN-A2 (PTHR14167:SF63)		0.02688	3.244213135
**Q14764**	MVP_HUMAN	Major vault protein;MVP;ortholog	MAJOR VAULT PROTEIN (PTHR14165:SF3)	scaffold/adaptor protein(PC00226)	0.02776	3.309321869
**Q9BWD1**	THIC_HUMAN	Acetyl-CoA acetyltransferase, cytosolic;ACAT2;ortholog	ACETYL-COA ACETYLTRANSFERASE, CYTOSOLIC (PTHR18919:SF81)	acyltransferase(PC00042)	0.02855	1.842583088

**Q92597**	NDRG1_HUMAN	Protein NDRG1;NDRG1;ortholog	PROTEIN NDRG1 (PTHR11034:SF18)	serine protease(PC00203)	0.03241	2.897734821
**Q9UNN8**	EPCR_HUMAN	Endothelial protein C receptor;PROCR;ortholog	ENDOTHELIAL PROTEIN C RECEPTOR (PTHR15349:SF0)	protein-binding activity modulator(PC00095)	0.03415	2.22795871
**P69905**	HBA_HUMAN	Hemoglobin subunit alpha;HBA2;ortholog	HEMOGLOBIN SUBUNIT ALPHA (PTHR11442:SF48)		0.03428	4.58530993
**P11021**	BIP_HUMAN	Endoplasmic reticulum chaperone BiP;HSPA5;ortholog	ENDOPLASMIC RETICULUM CHAPERONE BIP (PTHR19375:SF380)		0.03429	1.884856296
**Q6X4U4**	SOSD1_HUMAN	Sclerostin domain-containing protein 1;SOSTDC1;ortholog	SCLEROSTIN DOMAIN-CONTAINING PROTEIN 1 (PTHR14903:SF5)	intercellular signal molecule(PC00207)	0.03445	0.362794853
**P05204**	HMGN2_HUMAN	Non-histone chromosomal protein HMG-17;HMGN2;ortholog	NON-HISTONE CHROMOSOMAL PROTEIN HMG-17 (PTHR23087:SF13)	chromatin/chromatin-binding, or -regulatory protein(PC00077)	0.03665	0.681892896
**Q99714**	HCD2_HUMAN	3-hydroxyacyl-CoA dehydrogenase type-2;HSD17B10;ortholog	2,4-DIENOYL-COA REDUCTASE, MITOCHONDRIAL-RELATED (PTHR43658:SF8)	oxidoreductase(PC00176)	0.03678	3.63205505
**P49321**	NASP_HUMAN	Nuclear autoantigenic sperm protein;NASP;ortholog	NUCLEAR AUTOANTIGENIC SPERM PROTEIN (PTHR15081:SF1)	chaperone(PC00072)	0.03969	0.320389344
**Q9P258**	RCC2_HUMAN	Protein RCC2;RCC2;ortholog	PROTEIN RCC2 (PTHR46207:SF1)		0.04374	1.821171165
**P29279**	CTGF_HUMAN	CCN family member 2;CCN2;ortholog	CCN FAMILY MEMBER 2 (PTHR11348:SF7)	growth factor(PC00112)	0.04517	1.892568606

Table S2: Differential expressed proteins in sEVs derived from 1% CAOV-3 cells in compared to exosomes derived from 8% CAOV-3 cells.

Mapped Ids	Peak Name	Gene Name/ Gene Symbol/ Ortholog	PANTHER Family /Subfamily	Pather Protein Class	p-value	Fold Change
**P08697**	A2AP_HUMAN	Alpha-2-antiplasmin;SERPINF2;ortholog	ALPHA-2-ANTIPLASMIN (PTHR11461:SF354)	protease inhibitor(PC00191)	0.00083	4.08827906
**P01008**	ANT3_HUMAN	Antithrombin-III;SERPINC1;ortholog	ANTITHROMBIN-III (PTHR11461:SF53)	protease inhibitor(PC00191)	0.00089	3.72433162
**P05141**	ADT2_HUMAN	ADP/ATP translocase 2;SLC25A5;ortholog	ADP/ATP TRANSLOCASE 2 (PTHR45635:SF3)	transfer/carrier protein(PC00219)	0.00161	4.82068417
**P54920**	SNAA_HUMAN	Alpha-soluble NSF attachment protein;NAPA;ortholog	ALPHA-SOLUBLE NSF ATTACHMENT PROTEIN (PTHR13768:SF23)	membrane traffic protein(PC00150)	0.00253	3.78320567
**Q9BWD1**	THIC_HUMAN	Protein unc-45 homolog A;UNC45A;ortholog	PROTEIN UNC-45 HOMOLOG A (PTHR45994:SF3)		0.00274	2.74911427
**P10909**	CLUS_HUMAN	Clusterin;CLU;ortholog	CLUSTERIN (PTHR10970:SF1)		0.0031	3.8284968
**P11766**	ADHX_HUMAN	Alcohol dehydrogenase class-3;ADH5;ortholog	ALCOHOL DEHYDROGENASE CLASS-3 (PTHR43880:SF4)	dehydrogenase(PC00092)	0.00392	2.17968741
**P01130**	LDLR_HUMAN	Low-density lipoprotein receptor;LDLR;ortholog	LOW-DENSITY LIPOPROTEIN RECEPTOR (PTHR24270:SF21)	apolipoprotein(PC00052)	0.00401	2.13165456
**Q04837**	SSBP_HUMAN	Bcl-2-like protein 1;BCL2L1;ortholog	BCL-2-LIKE PROTEIN 1 (PTHR11256:SF12)		0.00478	0.56010098
**P08727**	K1C19_HUMAN	Keratin, type I cytoskeletal 19;KRT19;ortholog	KERATIN, TYPE I CYTOSKELETAL 19 (PTHR23239:SF14)		0.00514	12.4300286
**Q99538**	LGMN_HUMAN	Aconitate hydratase, mitochondrial;ACO2;ortholog	ACONITATE HYDRATASE, MITOCHONDRIAL (PTHR43160:SF6)	hydratase(PC00120)	0.00553	6.86414084
**P46779**	RL28_HUMAN	60S ribosomal protein L28;RPL28;ortholog	60S RIBOSOMAL PROTEIN L28 (PTHR10544:SF12)	ribosomal protein(PC00202)	0.00591	0.20440704
**P54819**	KAD2_HUMAN	Adenylate kinase 2, mitochondrial;AK2;ortholog	ADENYLATE KINASE 2, MITOCHONDRIAL (PTHR23359:SF227)	nucleotide kinase(PC00172)	0.00616	7.88524548
**P23368**	MAOM_HUMAN	NAD-dependent malic enzyme, mitochondrial;ME2;ortholog	NAD-DEPENDENT MALIC ENZYME, MITOCHONDRIAL (PTHR23406:SF27)	oxidoreductase(PC00176)	0.0067	2.93587079
**P62081**	RS7_HUMAN	40S ribosomal protein S7;RPS7;ortholog	40S RIBOSOMAL PROTEIN S7 (PTHR11278:SF5)	ribosomal protein(PC00202)	0.00925	0.13344482
**Q15800**	MSMO1_HUMAN	26S proteasome non-ATPase regulatory subunit 5;PSMD5;ortholog	26S PROTEASOME NON-ATPASE REGULATORY SUBUNIT 5 (PTHR13554:SF10)	protease(PC00190)	0.00964	3.10769602
**P08962**	CD63_HUMAN	CD63 antigen;CD63;ortholog	CD63 ANTIGEN (PTHR19282:SF471)		0.01208	2.57763862
**P00734**	THRB_HUMAN	Prothrombin;F2;ortholog	PROTHROMBIN (PTHR24254:SF10)	serine protease(PC00203)	0.01218	3.75581841
**O75822**	EIF3J_HUMAN	Eukaryotic translation initiation factor 3 subunit J;EIF3J;ortholog	EUKARYOTIC TRANSLATION INITIATION FACTOR 3 SUBUNIT J (PTHR21681:SF0)	translation initiation factor(PC00224)	0.01281	12.3470777
**Q8NG11**	TSN14_HUMAN	Programmed cell death 6-interacting protein;PDCD6IP;ortholog	PROGRAMMED CELL DEATH 6-INTERACTING PROTEIN (PTHR23030:SF39)	membrane traffic protein(PC00150)	0.01447	2.69442195
**Q8N1G4**	LRC47_HUMAN	Tetraspanin-14;TSPAN14;ortholog	TETRASPANIN-14 (PTHR19282:SF261)		0.01484	5.74951622

**Q13263**	TIF1B_HUMAN	Dynactin subunit 2;DCTN2;ortholog	DYNACTIN SUBUNIT 2 (PTHR15346:SF0)	microtubule binding motor protein(PC00156)	0.0155	17.6460407
**Q9UQ80**	PA2G4_HUMAN	RuvB-like 1;RUVBL1;ortholog	RUVB-LIKE 1 (PTHR11093:SF6)		0.01561	0.18970385
**P11047**	LAMC1_HUMAN	Laminin subunit gamma-1;LAMC1;ortholog	LAMININ SUBUNIT GAMMA-1 (PTHR10574:SF270)	extracellular matrix protein(PC00102)	0.01589	2.83518596
**Q9UNF1**	MAGD2_HUMAN	Proliferation-associated protein 2G4;PA2G4;ortholog	PROLIFERATION-ASSOCIATED PROTEIN 2G4 (PTHR10804:SF130)	protease(PC00190)	0.01767	2.1957425
**Q8WUM4**	PDC6I_HUMAN	Legumain;LGMN;ortholog	LEGUMAIN (PTHR12000:SF38)	cysteine protease(PC00081)	0.01785	2.43214344
**P14209**	CD99_HUMAN	CD99 antigen;CD99;ortholog	CD99 ANTIGEN (PTHR15076:SF15)		0.0192	2.75028881
**P13995**	MTDC_HUMAN	Bifunctional methylenetetrahydrofolate dehydrogenase/cyclohydrolase, mitochondrial;MTHFD2;ortholog	BIFUNCTIONAL METHYLENETETRAHYDROFOLATE DEHYDROGENASE/CYCLOHYDROLASE, MITOCHONDRIAL (PTHR48099:SF15)	0.01934	4.38682582	0.64215039
**P43243**	MATR3_HUMAN	Matrin-3;MATR3;ortholog	MATRIN-3 (PTHR15592:SF2)		0.02346	2.16067507
**O94826**	TOM70_HUMAN	Mitochondrial import receptor subunit TOM70;TOMM70;ortholog	MITOCHONDRIAL IMPORT RECEPTOR SUBUNIT TOM70 (PTHR46208:SF1)	primary active transporter(PC00068)	0.02357	0.34372307
**Q9H3U1**	UN45A_HUMAN	Septin-9;SEPTIN9;ortholog	SEPTIN-9 (PTHR18884:SF47)	cytoskeletal protein(PC00085)	0.02394	3.27024591
**Q9UHD8**	SEPT9_HUMAN	Multifunctional methyltransferase subunit TRM112-like protein;TRMT112;ortholog	MULTIFUNCTIONAL METHYLTRANSFERASE SUBUNIT TRM112-LIKE PROTEIN (PTHR12773:SF2)	0.02496	1.34614271	0.1290911
**O95782**	AP2A1_HUMAN	AP-2 complex subunit alpha-1;AP2A1;ortholog	AP-2 COMPLEX SUBUNIT ALPHA-1 (PTHR22780:SF33)	membrane traffic protein(PC00150)	0.02496	2.58909383
**Q9BRF8**	CPPED_HUMAN	Acetyl-CoA acetyltransferase, cytosolic;ACAT2;ortholog	ACETYL-COA ACETYLTRANSFERASE, CYTOSOLIC (PTHR18919:SF81)	acyltransferase(PC00042)	0.02553	3.55474749
**Q9UI30**	TR112_HUMAN	Proteasome activator complex subunit 2;PSME2;ortholog	PROTEASOME ACTIVATOR COMPLEX SUBUNIT 2 (PTHR10660:SF6)		0.02636	4.1208149
**P01024**	CO3_HUMAN	Complement C3;C3;ortholog	COMPLEMENT C3 (PTHR11412:SF81)	protease inhibitor(PC00191)	0.02773	4.41240783
**O00232**	PSD12_HUMAN	26S proteasome non-ATPase regulatory subunit 12;PSMD12;ortholog	26S PROTEASOME NON-ATPASE REGULATORY SUBUNIT 12 (PTHR10855:SF1)	protease(PC00190)	0.02787	0.19155232
**Q9UL46**	PSME2_HUMAN	Melanoma-associated antigen D2;MAGED2;ortholog	MELANOMA-ASSOCIATED ANTIGEN D2 (PTHR11736:SF11)	scaffold/adaptor protein(PC00226)	0.02812	3.93921806
**P42704**	LPPRC_HUMAN	Leucine-rich PPR motif-containing protein, mitochondrial;LRPPRC;ortholog	LEUCINE-RICH PPR MOTIF-CONTAINING PROTEIN, MITOCHONDRIAL (PTHR46669:SF1)	0.02832	2.50153002	0.39820572
**Q13561**	DCTN2_HUMAN	Nidogen-2;NID2;ortholog	NIDOGEN-2 (PTHR12352:SF3)	calmodulin-related(PC00061)	0.02846	9.68919945
**Q14112**	NID2_HUMAN	Methylsterol monooxygenase 1;MSMO1;ortholog	METHYLSTEROL MONOOXYGENASE 1 (PTHR11863:SF3)	oxidase(PC00175)	0.02956	2.80446616
**Q6WKZ4**	RFIP1_HUMAN	Histone H3.2;H3C14;ortholog	HISTONE H3.2 (PTHR11426:SF232)	histone(PC00118)	0.02963	4.20208778
**Q13126**	MTAP_HUMAN	Transcription intermediary factor 1-beta;TRIM28;ortholog	TRANSCRIPTION INTERMEDIARY FACTOR 1-BETA (PTHR25462:SF274)		0.03141	4.80223729
**Q16401**	PSMD5_HUMAN	Hexokinase HKDC1;HKDC1;ortholog	HEXOKINASE HKDC1 (PTHR19443:SF28)	kinase(PC00137)	0.03233	3.3350955

**Q09666**	AHNK_HUMAN	S-methyl-5'-thioadenosine phosphorylase;MTAP;ortholog	S-METHYL-5'-THIOADENOSINE PHOSPHORYLASE (PTHR42679:SF4)	nucleotide kinase(PC00172)	0.03254	3.16204907
**Q9Y265**	RUVB1_HUMAN	RuvB-like 1, RUVBL1, ortholog	RUVB-LIKE 1 (PTHR11093:SF6)		0.03261	2.38764834
**Q07817**	B2CL1_HUMAN	Neuroblast differentiation-associated protein AHNAK;AHNAK;ortholog	NEUROBLAST DIFFERENTIATION-ASSOCIATED PROTEIN AHNAK (PTHR23348:SF41)	0.03295	2.46209558	0.39130491
**Q2TB90**	HKDC1_HUMAN	Rab11 family-interacting protein 1;RAB11FIP1;ortholog	RAB11 FAMILY-INTERACTING PROTEIN 1 (PTHR15746:SF22)		0.03598	3.82367919
**O00170**	AIP_HUMAN	AH receptor-interacting protein;AIP;ortholog	AH RECEPTOR-INTERACTING PROTEIN (PTHR11242:SF3)	chaperone(PC00072)	0.03726	2.65536185
**P19224**	UD16_HUMAN	UDP-glucuronosyltransferase 1-6;UGT1A6;ortholog	UDP-GLUCURONOSYLTRANSFERASE 1-6 (PTHR48050:SF7)	glycosyltransferase(PC00111)	0.03841	5.3263806
**Q71DI3**	H32_HUMAN	Leucine-rich repeat-containing protein 47;LRRC47;ortholog	LEUCINE-RICH REPEAT-CONTAINING PROTEIN 47 (PTHR10947:SF3)	aminoacyl-tRNA synthetase(PC00047)	0.03847	0.07189331
**P02749**	APOH_HUMAN	Beta-2-glycoprotein 1;APOH;ortholog	BETA-2-GLYCOPROTEIN 1 (PTHR19325:SF549)		0.03894	5.85732831
**P19823**	ITIH2_HUMAN	Inter-alpha-trypsin inhibitor heavy chain H2;ITIH2;ortholog	INTER-ALPHA-TRYPSIN INHIBITOR HEAVY CHAIN H2 (PTHR10338:SF14)	protease inhibitor(PC00191)	0.03921	4.87285
**P34897**	GLYM_HUMAN	Serine hydroxymethyltransferase, mitochondrial;SHMT2;ortholog	SERINE HYDROXYMETHYLTRANSFERASE, MITOCHONDRIAL (PTHR11680:SF53)	methyltransferase(PC00155)	0.04049	0.47259358
**P43304**	GPDM_HUMAN	Glycerol-3-phosphate dehydrogenase, mitochondrial;GPD2;ortholog	GLYCEROL-3-PHOSPHATE DEHYDROGENASE, MITOCHONDRIAL (PTHR11985:SF29)	dehydrogenase(PC00092)	0.04072	5.20909443
**Q99798**	ACON_HUMAN	Serine/threonine-protein phosphatase CPPED1;CPPED1;ortholog	SERINE/THREONINE-PROTEIN PHOSPHATASE CPPED1 (PTHR43143:SF1)		0.0408	2.64302805
**P60842**	IF4A1_HUMAN	Eukaryotic initiation factor 4A-I;EIF4A1;ortholog	EUKARYOTIC INITIATION FACTOR 4A-I (PTHR24031:SF221)		0.04115	2.06622701
**P51659**	DHB4_HUMAN	Peroxisomal multifunctional enzyme type 2;HSD17B4;ortholog	PEROXISOMAL MULTIFUNCTIONAL ENZYME TYPE 2 (PTHR13078:SF56)		0.04359	9.17935303
**P41252**	SYIC_HUMAN	Isoleucine--tRNA ligase, cytoplasmic;IARS1;ortholog	ISOLEUCINE--TRNA LIGASE, CYTOPLASMIC (PTHR42780:SF1)	aminoacyl-tRNA synthetase(PC00047)	0.04482	2.01765737
**O95336**	6PGL_HUMAN	6-phosphogluconolactonase;PGLS;ortholog	6-PHOSPHOGLUCONOLACTONASE (PTHR11054:SF0)	hydrolase(PC00121)	0.04518	2.64133289
**P17812**	PYRG1_HUMAN	CTP synthase 1;CTPS1;ortholog	CTP SYNTHASE 1 (PTHR11550:SF8)	ligase(PC00142)	0.04528	5.42056117
**P11908**	PRPS2_HUMAN	Ribose-phosphate pyrophosphokinase 2;PRPS2;ortholog	RIBOSE-PHOSPHATE PYROPHOSPHOKINASE 2 (PTHR10210:SF110)	nucleotide kinase(PC00172)	0.04566	3.54000103
**P49411**	EFTU_HUMAN	Elongation factor Tu, mitochondrial;TUFM;ortholog	ELONGATION FACTOR TU, MITOCHONDRIAL (PTHR43721:SF22)	translation elongation factor(PC00222)	0.04845	4.28367016
**Q00325-2**	MPCP_HUMAN	Single-stranded DNA-binding protein, mitochondrial;SSBP1;ortholog	SINGLE-STRANDED DNA-BINDING PROTEIN, MITOCHONDRIAL (PTHR10302:SF0)	DNA metabolism protein(PC00009)	0.04862	4.70816339
**O15355**	PPM1G_HUMAN	Protein phosphatase 1G;PPM1G;ortholog	PROTEIN PHOSPHATASE 1G (PTHR13832:SF805)	protein phosphatase(PC00195)	0.04977	1.96627827

Table S3. A comparison of proteins differentially expressed in 1% O_2 sEVs and 8% O_2 sEVs.

Protein	p-value	Fold change
Glycolysis related proteins		
Hexokinase (HK)	0.036	3.82
UDP-glucuronosyltransferase 1-6 (UD16)	0.038	5.33
6-phosphogluconolactonase (6PGL)	0.045	2.64
CTP synthase 1 (PYRG1)	0.045	5.42
Detoxification associated proteins		
Alcohol dehydrogenase class-3 (ADH5)	0.004	2.18
NAD-dependent malic enzyme (MAMO)	0.007	2.93
Bifunctional methylenetetrahydrofolate dehydrogenase/cyclohydrolase (MTDC)	0.019	4.39
Peroxisomal multifunctional enzyme type 2 (DHB4)	0.044	9.18

Table S4. Target Set of Metabolic and Reference Proteins.

Pathways	Peptides	Protein code
**Main glycolysis pathway**	NLVTEDVMR	GPI
	SNTPILVDGK	GPI
	TFTTQETITNAETAK	GPI
	GQLESIVENIR	PFKL
	EWSGLLEELAR	PFKP
	TFVLEVMGR	PFKP
	YLEEIATQMR	PFKP
	ALANSLACQGK	ALDO
	QLLLTADDR	ALDO
	ELSDIAHR	ALDOA
	GILAADESTGSIAK	ALDOA
	LQSIGTENTEENR	ALDOA
	HVFGESDELIGQK	TP1
	IIYGGSVTGATCK	TP1
	VVLAYEPVWAIGTGK	TP1
	FFVGGNWK	TPI
	QSLGELIGTLNAAK	TPI
	TATPQQAQEVHEK	TPI
	VVFEQTK	TPI
	IISNASCTTNCLAPLAK	GAPDH
	VPTANVSVVDLTCR	GAPDH
	ALMDEVVK	PGK1
	ELNYFAK	PGK1

	LGDVYVNDAFGTAHR	PGK1
	VDFNVPMK	PGK1
	VSHVSTGGGASLELLEGK	PGK1
	AHSSMVGVNLPQK	PGK1
	ALPFWNEEIVPQIK	PGAM
	YADLTEDQLPSCESLK	PGAM1
	TIAPALVSK	ENO
	VVIGMDVAASEFFR	ENO
	AAVPSGASTGIYEALELR	ENO1
	LMIEMDGTENK	ENO1
	VNQIGSVTESLQACK	ENO1
	YISPDQLADLYK	ENO1
	CCSGAIIVLTK	PKM2
	GDLGIEIPAEK	PKM2
	GDYPLEAVR	PKM2
	GIFPVLCK	PKM2
	GSGTAEVELK	PKM2
	ITLDNAYMEK	PKM2
	IYVDDGLISLQVK	PKM2
	LDIDSPPITAR	PKM2
	NTGIICTIGPASR	PKM2
**Glycolysis branching pathways**	FIIPNVVK	LDHA
	LVIITAGAR	LDHA
	QVVESAYEVIK	LDHA
	SLADELALVDVLEDK	LDHA
	VIGSGCNLDSAR	LDHA

	GGYFDEFGIIR	G6PD
	GPTEADELMK	G6PD
	GYLDDPTVPR	G6PD
	HQPTAIIAK	TKT
	NSTFSEIFK	TKT
**Other metabolic proteins**	AALQEELQLCK	FASN
	ACLDTAVENMPSLK	FASN
	AQVADVVVSR	FASN
	SLLVNPEGPTLMR	FASN
	VLEALLPLK	FASN
	FVEGLPINDFSR	MDH1
	GEFVTTVQQR	MDH1
**Reference proteins**	GYSFTTTAER	ACTAB
	HQGVMVGMGQK	ACTAB
	QEYDESGPSIVHR	ACTAB
	AGFAGDDAPR	ACTAB
	EITALAPSTMK	ACTAB
	IIQLLDDYPK	RLPL0
	EGMNIVEAMER	PPIA
	FEDENFILK	PPIA
	IIPGFMCQGGDFTR	PPIA
	FLIPNASQAESK	YWHAZ

Table S5. Differentially expressed proteins in cells incubated with hypoxic exosomes compared to cells incubated with normoxic sEVs

Mapped Ids	Peak Name	Gene Name/ Gene Symbol/ Ortholog	PANTHER Family /Subfamily	Pather Protein Class	p-value	Fold Change
**O43242**	RS14_HUMAN	40S ribosomal protein S14;RPS14;ortholog	40S RIBOSOMAL PROTEIN S14 (PTHR11759:SF58)	ribosomal protein(PC00202)	0.00000227	0.267584
**P08670**	COMP_HUMAN	Cartilage oligomeric matrix protein;COMP;ortholog	CARTILAGE OLIGOMERIC MATRIX PROTEIN (PTHR10199:SF88)	NA	0.00024	0.765258
**P55036**	HSPB1_HUMAN	Heat shock protein beta-1;HSPB1;ortholog	HEAT SHOCK PROTEIN BETA-1 (PTHR45640:SF7)	NA	0.00041	1.131776
**P45880**	ECI1_HUMAN	Enoyl-CoA delta isomerase 1, mitochondrial;ECI1;ortholog	ENOYL-COA DELTA ISOMERASE 1, MITOCHONDRIAL (PTHR11941:SF45)	hydratase(PC00120)	0.00055	0.879293
**P40429**	RL5_HUMAN	60S ribosomal protein L5;RPL5;ortholog	60S RIBOSOMAL PROTEIN L5 (PTHR23410:SF24)	ribosomal protein(PC00202)	0.0006	1.231754
**Q86VP6**	EPCR_HUMAN	Endothelial protein C receptor;PROCR;ortholog	ENDOTHELIAL PROTEIN C RECEPTOR (PTHR15349:SF0)	protein-binding activity modulator(PC00095)	0.00066	0.695279
**Q06481**	ALDOC_HUMAN	Fructose-bisphosphate aldolase C;ALDOC;ortholog	FRUCTOSE-BISPHOSPHATE ALDOLASE C (PTHR11627:SF3)	aldolase(PC00044)	0.00067	1.664374
**P49588**	VDAC2_HUMAN	Voltage-dependent anion-selective channel protein 2;VDAC2;ortholog	VOLTAGE-DEPENDENT ANION-SELECTIVE CHANNEL PROTEIN 2 (PTHR11743:SF12)	voltage-gated ion channel(PC00241)	0.00077	1.15718
**P78347**	COPB_HUMAN	Coatomer subunit beta;COPB1;ortholog	COATOMER SUBUNIT BETA (PTHR10635:SF0)	vesicle coat protein(PC00235)	0.00081	0.796536
**Q9UJS0**	NPM_HUMAN	Nucleophosmin;NPM1;ortholog	NUCLEOPHOSMIN (PTHR22747:SF28)	chaperone(PC00072)	0.00091	0.857056
**Q9NR96**	RS8_HUMAN	40S ribosomal protein S8;RPS8;ortholog	40S RIBOSOMAL PROTEIN S8 (PTHR10394:SF12)	ribosomal protein(PC00202)	0.00112	1.139647
**Q6X4U4**	RL28_HUMAN	60S ribosomal protein L28;RPL28;ortholog	60S RIBOSOMAL PROTEIN L28 (PTHR10544:SF12)	ribosomal protein(PC00202)	0.00117	1.247209
**P11279**	RAB32_HUMAN	Ras-related protein Rab-32;RAB32;ortholog	RAS-RELATED PROTEIN RAB-32 (PTHR24073:SF1176)	NA	0.00125	1.258191
**P09496**	PARP1_HUMAN	Poly [ADP-ribose] polymerase 1;PARP1;ortholog	POLY [ADP-RIBOSE] POLYMERASE 1 (PTHR10459:SF112)	DNA metabolism protein(PC00009)	0.00126	0.870104
**P53602**	KCC2D_HUMAN	Calcium/calmodulin-dependent protein kinase type II subunit delta;CAMK2D;ortholog	CALCIUM/CALMODULIN-DEPENDENT PROTEIN KINASE TYPE II SUBUNIT DELTA (PTHR24347:SF365)	non-receptor serine/threonine protein kinase(PC00167)	0.00183	1.23107
**Q8N163**	PUR6_HUMAN	Multifunctional protein ADE2;PAICS;ortholog	MULTIFUNCTIONAL PROTEIN ADE2 (PTHR43599:SF3)	NA	0.00253	1.129759

**Q13620**	COPD_HUMAN	Coatomer subunit delta;ARCN1;ortholog	COATOMER SUBUNIT DELTA (PTHR10121:SF0)	vesicle coat protein(PC00235)	0.00287	0.723674
**P19105**	CMC2_HUMAN	Calcium-binding mitochondrial carrier protein Aralar2;SLC25A13;ortholog	CALCIUM-BINDING MITOCHONDRIAL CARRIER PROTEIN ARALAR2 (PTHR45678:SF12)	secondary carrier transporter(PC00258)	0.00288	1.27725
**P11169**	HMCS1_HUMAN	Hydroxymethylglutaryl-CoA synthase, cytoplasmic;HMGCS1;ortholog	HYDROXYMETHYLGLUTARYL-COA SYNTHASE, CYTOPLASMIC (PTHR43323:SF4)	NA	0.00341	0.76832
**P51858**	IMA3_HUMAN	Importin subunit alpha-3;KPNA4;ortholog	IMPORTIN SUBUNIT ALPHA-3 (PTHR23316:SF7)	transporter(PC00227)	0.00372	3.844421
**O00767**	1433B_HUMAN	14-3-3 protein beta/alpha;YWHAB;ortholog	14-3-3 PROTEIN BETA/ALPHA (PTHR18860:SF28)	scaffold/adaptor protein(PC00226)	0.00383	0.857004
**P09874**	RU2A_HUMAN	U2 small nuclear ribonucleoprotein A';SNRPA1;ortholog	U2 SMALL NUCLEAR RIBONUCLEOPROTEIN A' (PTHR10552:SF6)	RNA splicing factor(PC00148)	0.00418	0.895571
**P42126**	RB11B_HUMAN	Ras-related protein Rab-11B;RAB11B;ortholog	RAS-RELATED PROTEIN RAB-11B (PTHR47979:SF41)	NA	0.00429	0.910052
**P53999**	TRA2B_HUMAN	Transformer-2 protein homolog beta;TRA2B;ortholog	TRANSFORMER-2 PROTEIN HOMOLOG BETA (PTHR48034:SF1)	NA	0.00435	0.350914
**P04181**	SYRC_HUMAN	Arginine--tRNA ligase, cytoplasmic;RARS1;ortholog	ARGININE--TRNA LIGASE, CYTOPLASMIC (PTHR11956:SF5)	aminoacyl-tRNA synthetase(PC00047)	0.00453	0.773548
**P22234**	CH60_HUMAN	60 kDa heat shock protein, mitochondrial;HSPD1;ortholog	60 KDA HEAT SHOCK PROTEIN, MITOCHONDRIAL (PTHR45633:SF17)	NA	0.00479	0.779151
**P62263**	ROA2_HUMAN	Heterogeneous nuclear ribonucleoproteins A2/B1;HNRNPA2B1;ortholog	HETEROGENEOUS NUCLEAR RIBONUCLEOPROTEINS A2/B1 (PTHR48026:SF13)	NA	0.00547	0.902825
**Q13200**	GCP2_HUMAN	Gamma-tubulin complex component 2;TUBGCP2;ortholog	GAMMA-TUBULIN COMPLEX COMPONENT 2 (PTHR19302:SF13)	non-motor microtubule binding protein(PC00166)	0.00547	0.779102
**Q01581**	CAND1_HUMAN	Cullin-associated NEDD8-dissociated protein 1;CAND1;ortholog	CULLIN-ASSOCIATED NEDD8-DISSOCIATED PROTEIN 1 (PTHR12696:SF1)	ubiquitin-protein ligase(PC00234)	0.0055	0.918307
**O15269**	TALDO_HUMAN	Transaldolase;TALDO1;ortholog	TRANSALDOLASE (PTHR10683:SF18)	aldolase(PC00044)	0.00629	1.211286
**Q92598**	COX2_HUMAN	Cytochrome c oxidase subunit 2;MT-CO2;ortholog	CYTOCHROME C OXIDASE SUBUNIT 2 (PTHR22888:SF9)	oxidoreductase(PC00176)	0.00652	0.791912
**P11142**	OAT_HUMAN	Ornithine aminotransferase, mitochondrial;OAT;ortholog	ORNITHINE AMINOTRANSFERASE, MITOCHONDRIAL (PTHR11986:SF118)	transaminase(PC00216)	0.00733	1.162815
**Q96M27**	RS6_HUMAN	40S ribosomal protein S6;RPS6;ortholog	40S RIBOSOMAL PROTEIN S6 (PTHR11502:SF33)	ribosomal protein(PC00202)	0.00788	1.301697
**Q9NR30**	GCSH_HUMAN	Glycine cleavage system H protein, mitochondrial;GCSH;ortholog	GLYCINE CLEAVAGE SYSTEM H PROTEIN, MITOCHONDRIAL (PTHR11715:SF28)	NA	0.00829	1.441947
**P53618**	CAH2_HUMAN	Carbonic anhydrase 2;CA2;ortholog	CARBONIC ANHYDRASE 2 (PTHR18952:SF120)	NA	0.0084	1.224134
**O60762**	MVD1_HUMAN	Diphosphomevalonate decarboxylase;MVD;ortholog	DIPHOSPHOMEVALONATE DECARBOXYLASE (PTHR10977:SF3)	decarboxylase(PC00089)	0.00865	0.730017

**O75533**	RL23_HUMAN	60S ribosomal protein L23;RPL23;ortholog	60S RIBOSOMAL PROTEIN L23 (PTHR11761:SF22)	ribosomal protein(PC00202)	0.00905	0.90547
**P26373**	PFKAL_HUMAN	ATP-dependent 6-phosphofructokinase, liver type;PFKL;ortholog	ATP-DEPENDENT 6-PHOSPHOFRUCTOKINASE, LIVER TYPE (PTHR13697:SF14)	carbohydrate kinase(PC00065)	0.00906	0.669557
**Q16186**	SPTC1_HUMAN	Serine palmitoyltransferase 1;SPTLC1;ortholog	SERINE PALMITOYLTRANSFERASE 1 (PTHR13693:SF2)	transaminase(PC00216)	0.00911	1.085099
**Q9NSD9**	CLCA_HUMAN	Clathrin light chain A;CLTA;ortholog	CLATHRIN LIGHT CHAIN A (PTHR10639:SF1)	vesicle coat protein(PC00235)	0.00927	0.815368
**P49327**	HM13_HUMAN	Minor histocompatibility antigen H13;HM13;ortholog	MINOR HISTOCOMPATIBILITY ANTIGEN H13 (PTHR12174:SF23)	aspartic protease(PC00053)	0.01001	1.18603
**Q12874**	PGBM_HUMAN	Basement membrane-specific heparan sulfate proteoglycan core protein;HSPG2;ortholog	BASEMENT MEMBRANE-SPECIFIC HEPARAN SULFATE PROTEOGLYCAN CORE PROTEIN (PTHR10574:SF273)	extracellular matrix protein(PC00102)	0.01079	1.14086
**P62753**	GTR3_HUMAN	Solute carrier family 2, facilitated glucose transporter member 3;SLC2A3;ortholog	SOLUTE CARRIER FAMILY 2, FACILITATED GLUCOSE TRANSPORTER MEMBER 3 (PTHR23503:SF99)	NA	0.01171	0.886411
**P09661**	NDRG1_HUMAN	Protein NDRG1;NDRG1;ortholog	PROTEIN NDRG1 (PTHR11034:SF18)	serine protease(PC00203)	0.01195	0.811879
**P83731**	SYSC_HUMAN	Serine--tRNA ligase, cytoplasmic;SARS1;ortholog	SERINE--TRNA LIGASE, CYTOPLASMIC-RELATED (PTHR11778:SF7)	aminoacyl-tRNA synthetase(PC00047)	0.01277	0.682187
**Q9NS69**	VIME_HUMAN	Vimentin;VIM;ortholog	VIMENTIN (PTHR45652:SF5)	NA	0.01328	0.684403
**P22626**	1433G_HUMAN	14-3-3 protein gamma;YWHAG;ortholog	14-3-3 PROTEIN GAMMA (PTHR18860:SF22)	scaffold/adaptor protein(PC00226)	0.01357	1.061605
**Q9UBM7**	DHB12_HUMAN	Very-long-chain 3-oxoacyl-CoA reductase;HSD17B12;ortholog	VERY-LONG-CHAIN 3-OXOACYL-COA REDUCTASE (PTHR43899:SF14)	NA	0.01437	0.337966
**P31946**	PRS6A_HUMAN	26S proteasome regulatory subunit 6A;PSMC3;ortholog	26S PROTEASOME REGULATORY SUBUNIT 6A (PTHR23073:SF90)	protease(PC00190)	0.01479	0.911007
**P53801**	PLEC_HUMAN	Plectin;PLEC;ortholog	PLECTIN (PTHR23169:SF20)	intermediate filament binding protein(PC00130)	0.01513	1.214068
**P61221**	UGPA_HUMAN	UTP--glucose-1-phosphate uridylyltransferase;UGP2;ortholog	UTP--GLUCOSE-1-PHOSPHATE URIDYLYLTRANSFERASE (PTHR43511:SF4)	NA	0.01515	1.244052
**P49736**	SF3B1_HUMAN	Splicing factor 3B subunit 1;SF3B1;ortholog	SPLICING FACTOR 3B SUBUNIT 1 (PTHR12097:SF0)	RNA splicing factor(PC00148)	0.01517	1.095972
**Q15149**	SOSD1_HUMAN	Sclerostin domain-containing protein 1;SOSTDC1;ortholog	SCLEROSTIN DOMAIN-CONTAINING PROTEIN 1 (PTHR14903:SF5)	intercellular signal molecule(PC00207)	0.0162	0.848756
**P46779**	ABCE1_HUMAN	ATP-binding cassette sub-family E member 1;ABCE1;ortholog	ATP-BINDING CASSETTE SUB-FAMILY E MEMBER 1 (PTHR19248:SF16)	NA	0.01628	0.867793
**Q9UBI6**	CUL4B_HUMAN	Cullin-4B;CUL4B;ortholog	CULLIN-4B (PTHR11932:SF66)	ubiquitin-protein ligase(PC00234)	0.01713	1.156406
**P00403**	COPZ1_HUMAN	Coatomer subunit zeta-1;COPZ1;ortholog	COATOMER SUBUNIT ZETA-1 (PTHR11043:SF2)	vesicle coat protein(PC00235)	0.01721	0.888619
**P13726**	RL35A_HUMAN	60S ribosomal protein L35a;RPL35A;ortholog	60S RIBOSOMAL PROTEIN L35A (PTHR10902:SF30)	ribosomal protein(PC00202)	0.01739	0.688932

**P16949**	EIF3H_HUMAN	Eukaryotic translation initiation factor 3 subunit H;EIF3H;ortholog	EUKARYOTIC TRANSLATION INITIATION FACTOR 3 SUBUNIT H (PTHR10410:SF3)	translation initiation factor(PC00224)	0.01757	1.165479
**O15372**	UBP5_HUMAN	Ubiquitin carboxyl-terminal hydrolase 5;USP5;ortholog	UBIQUITIN CARBOXYL-TERMINAL HYDROLASE 5 (PTHR24006:SF655)	cysteine protease(PC00081)	0.01776	1.109794
**Q9NQ88**	LAMP1_HUMAN	Lysosome-associated membrane glycoprotein 1;LAMP1;ortholog	LYSOSOME-ASSOCIATED MEMBRANE GLYCOPROTEIN 1 (PTHR11506:SF27)	membrane trafficking regulatory protein(PC00151)	0.01795	1.120998
**Q04446**	MCM2_HUMAN	DNA replication licensing factor MCM2;MCM2;ortholog	DNA REPLICATION LICENSING FACTOR MCM2 (PTHR11630:SF44)	DNA metabolism protein(PC00009)	0.01818	0.932507
**P08134**	TIM44_HUMAN	Mitochondrial import inner membrane translocase subunit TIM44;TIMM44;ortholog	MITOCHONDRIAL IMPORT INNER MEMBRANE TRANSLOCASE SUBUNIT TIM44 (PTHR10721:SF1)	transporter(PC00227)	0.01894	0.811457
**P17980**	ROAA_HUMAN	Heterogeneous nuclear ribonucleoprotein A/B;HNRNPAB;ortholog	HETEROGENEOUS NUCLEAR RIBONUCLEOPROTEIN A/B (PTHR48033:SF1)	NA	0.01914	0.230408
**P49721**	CDC42_HUMAN	Cell division control protein 42 homolog;CDC42;ortholog	CELL DIVISION CONTROL PROTEIN 42 HOMOLOG (PTHR24072:SF136)	small GTPase(PC00208)	0.01927	1.062811
**Q9Y3A5**	DPM1_HUMAN	Dolichol-phosphate mannosyltransferase subunit 1;DPM1;ortholog	DOLICHOL-PHOSPHATE MANNOSYLTRANSFERASE SUBUNIT 1 (PTHR43398:SF1)	glycosyltransferase(PC00111)	0.0194	0.666285
**Q8TC12**	RL18A_HUMAN	60S ribosomal protein L18a;RPL18A;ortholog	60S RIBOSOMAL PROTEIN L18A (PTHR10052:SF50)	ribosomal protein(PC00202)	0.01969	0.682036
**P25205**	HEXI1_HUMAN	Protein HEXIM1;HEXIM1;ortholog	PROTEIN HEXIM1 (PTHR13469:SF7)	NA	0.02003	0.698776
**Q9HB07**	ACLY_HUMAN	ATP-citrate synthase;ACLY;ortholog	ATP-CITRATE SYNTHASE (PTHR23118:SF0)	transferase(PC00220)	0.02008	0.319368
**P17858**	TLR9_HUMAN	Toll-like receptor 9;TLR9;ortholog	TOLL-LIKE RECEPTOR 9 (PTHR47410:SF3)	transmembrane signal receptor(PC00197)	0.02017	0.391474
**P61923**	ACOD_HUMAN	Acyl-CoA desaturase;SCD;ortholog	ACYL-COA DESATURASE (PTHR11351:SF73)	NA	0.02051	0.749126
**Q9BXW7**	CGL_HUMAN	Cystathionine gamma-lyase;CTH;ortholog	CYSTATHIONINE GAMMA-LYASE (PTHR11808:SF15)	lyase(PC00144)	0.0213	0.22855
**P04792**	RRP1_HUMAN	Ribosomal RNA processing protein 1 homolog A;RRP1;ortholog	RIBOSOMAL RNA PROCESSING PROTEIN 1 HOMOLOG A (PTHR13026:SF1)	NA	0.02226	0.791443
**P49747**	SBDS_HUMAN	Ribosome maturation protein SBDS;SBDS;ortholog	RIBOSOME MATURATION PROTEIN SBDS (PTHR10927:SF1)	RNA metabolism protein(PC00031)	0.02296	0.659036
**Q99729**	SYFB_HUMAN	Phenylalanine--tRNA ligase beta subunit;FARSB;ortholog	PHENYLALANINE--TRNA LIGASE BETA SUBUNIT (PTHR10947:SF0)	aminoacyl-tRNA synthetase(PC00047)	0.02334	0.214782
**P62995**	PRRC1_HUMAN	Protein PRRC1;PRRC1;ortholog	PROTEIN PRRC1 (PTHR23276:SF2)	NA	0.0237	0.821451
**Q16851**	HS105_HUMAN	Heat shock protein 105 kDa;HSPH1;ortholog	HEAT SHOCK PROTEIN 105 KDA (PTHR45639:SF2)	NA	0.02479	1.133302
**Q07954**	SEP11_HUMAN	Septin-11;SEPTIN11;ortholog	SEPTIN-11 (PTHR18884:SF48)	cytoskeletal protein(PC00085)	0.02572	0.325575
**Q13557**	ARF4_HUMAN	ADP-ribosylation factor 4;ARF4;ortholog	ADP-RIBOSYLATION FACTOR 4 (PTHR11711:SF110)	G-protein(PC00020)	0.02602	1.166569

**P52789**	HDHD5_HUMAN	Haloacid dehalogenase-like hydrolase domain-containing 5;HDHD5;ortholog	HALOACID DEHALOGENASE-LIKE HYDROLASE DOMAIN-CONTAINING 5 (PTHR14269:SF17)	transferase(PC00220)	0.02732	0.896284
**Q9BSJ2**	DSG1_HUMAN	Desmoglein-1;DSG1;ortholog	DESMOGLEIN-1 (PTHR24025:SF9)	cadherin(PC00057)	0.02746	0.887029
**P18077**	MARE1_HUMAN	Microtubule-associated protein RP/EB family member 1;MAPRE1;ortholog	MICROTUBULE-ASSOCIATED PROTEIN RP/EB FAMILY MEMBER 1 (PTHR10623:SF20)	non-motor microtubule binding protein(PC00166)	0.02755	0.791303
**P56182**	UGDH_HUMAN	UDP-glucose 6-dehydrogenase;UGDH;ortholog	UDP-GLUCOSE 6-DEHYDROGENASE (PTHR11374:SF3)	dehydrogenase(PC00092)	0.02804	1.086134
**Q15907**	STMN1_HUMAN	Stathmin;STMN1;ortholog	STATHMIN (PTHR10104:SF5)	NA	0.02811	1.256738
**Q9UBN6**	LRP1_HUMAN	Prolow-density lipoprotein receptor-related protein 1;LRP1;ortholog	PROLOW-DENSITY LIPOPROTEIN RECEPTOR-RELATED PROTEIN 1 (PTHR24270:SF23)	apolipoprotein(PC00052)	0.02814	1.123403
**P10809**	HSP7C_HUMAN	Heat shock cognate 71 kDa protein;HSPA8;ortholog	HEAT SHOCK COGNATE 71 KDA PROTEIN (PTHR19375:SF379)	NA	0.02828	0.915755
**P62829**	PSB2_HUMAN	Proteasome subunit beta type-2;PSMB2;ortholog	PROTEASOME SUBUNIT BETA TYPE-2 (PTHR11599:SF6)	protease(PC00190)	0.02885	0.913065
**Q8TCT9**	G3BP1_HUMAN	Ras GTPase-activating protein-binding protein 1;G3BP1;ortholog	RAS GTPASE-ACTIVATING PROTEIN-BINDING PROTEIN 1 (PTHR10693:SF21)	RNA metabolism protein(PC00031)	0.02908	0.689067
**Q13283**	MYG1_HUMAN	MYG1 exonuclease;MYG1;ortholog	MYG1 EXONUCLEASE (PTHR11215:SF1)	hydrolase(PC00121)	0.02976	1.105623
**Q01082**	ML12A_HUMAN	Myosin regulatory light chain 12A;MYL12A;ortholog	MYOSIN REGULATORY LIGHT CHAIN 12A (PTHR23049:SF57)	actin or actin-binding cytoskeletal protein(PC00041)	0.03133	0.768661
**Q9UGI8**	APLP2_HUMAN	Amyloid-like protein 2;APLP2;ortholog	AMYLOID-LIKE PROTEIN 2 (PTHR23103:SF14)	protease inhibitor(PC00191)	0.03172	2.592006
**P53396**	ADRM1_HUMAN	Proteasomal ubiquitin receptor ADRM1;ADRM1;ortholog	PROTEASOMAL UBIQUITIN RECEPTOR ADRM1 (PTHR12225:SF0)	NA	0.03229	0.872436
**P06748**	ECH1_HUMAN	Delta(3,5)-Delta(2,4)-dienoyl-CoA isomerase, mitochondrial;ECH1;ortholog	DELTA(3,5)-DELTA(2,4)-DIENOYL-COA ISOMERASE, MITOCHONDRIAL (PTHR43149:SF1)	hydratase(PC00120)	0.03299	0.847672
**Q92575**	RDH11_HUMAN	Retinol dehydrogenase 11;RDH11;ortholog	RETINOL DEHYDROGENASE 11 (PTHR24320:SF108)	dehydrogenase(PC00092)	0.03303	1.186658
**O00629**	TIGAR_HUMAN	Fructose-2,6-bisphosphatase TIGAR;TIGAR;ortholog	FRUCTOSE-2,6-BISPHOSPHATASE TIGAR (PTHR46517:SF1)	phosphatase(PC00181)	0.03319	1.388604
**Q02543**	RL13A_HUMAN	60S ribosomal protein L13a;RPL13A;ortholog	60S RIBOSOMAL PROTEIN L13A (PTHR11545:SF30)	ribosomal protein(PC00202)	0.03326	0.594694
**P09972**	RL13_HUMAN	60S ribosomal protein L13;RPL13;ortholog	60S RIBOSOMAL PROTEIN L13 (PTHR11722:SF4)	ribosomal protein(PC00202)	0.03334	0.884622
**O94992**	GTF2I_HUMAN	General transcription factor II-I;GTF2I;ortholog	GENERAL TRANSCRIPTION FACTOR II-I (PTHR46304:SF2)	general transcription factor(PC00259)	0.03337	1.185212
**P98160**				NA	0.03486	0.761527

**Q92504**	PSMD4_HUMAN	26S proteasome non-ATPase regulatory subunit 4;PSMD4;ortholog	26S PROTEASOME NON-ATPASE REGULATORY SUBUNIT 4 (PTHR10223:SF7)	protease(PC00190)	0.03504	1.591623
**Q02413**	PTTG_HUMAN	Pituitary tumor-transforming gene 1 protein-interacting protein;PTTG1IP;ortholog	PITUITARY TUMOR-TRANSFORMING GENE 1 PROTEIN-INTERACTING PROTEIN (PTHR15191:SF2)	NA	0.03604	0.529322
**Q13637**	HNRPK_HUMAN	Heterogeneous nuclear ribonucleoprotein K;HNRNPK;ortholog	HETEROGENEOUS NUCLEAR RIBONUCLEOPROTEIN K (PTHR10288:SF307)	RNA metabolism protein(PC00031)	0.03713	0.472748
**Q13011**	PSMD3_HUMAN	26S proteasome non-ATPase regulatory subunit 3;PSMD3;ortholog	26S PROTEASOME NON-ATPASE REGULATORY SUBUNIT 3 (PTHR10758:SF2)	protease(PC00190)	0.03726	0.917002
**Q9UNN8**	CCAR2_HUMAN	Cell cycle and apoptosis regulator protein 2;CCAR2;ortholog	CELL CYCLE AND APOPTOSIS REGULATOR PROTEIN 2 (PTHR14304:SF12)	chromatin/chromatin-binding, or -regulatory protein(PC00077)	0.03743	2.514653
**O60701**	FAS_HUMAN	Fatty acid synthase;FASN;ortholog	FATTY ACID SYNTHASE (PTHR43775:SF7)	NA	0.03763	1.12561
**P61978**	TES_HUMAN	Testin;TES;ortholog	TESTIN (PTHR24211:SF1)	scaffold/adaptor protein(PC00226)	0.03775	1.164893
**O94826**	TCP4_HUMAN	Activated RNA polymerase II transcriptional coactivator p15;SUB1;ortholog	ACTIVATED RNA POLYMERASE II TRANSCRIPTIONAL COACTIVATOR P15 (PTHR13215:SF0)	general transcription factor(PC00259)	0.03811	0.621374
**P49591**	DHCR7_HUMAN	7-dehydrocholesterol reductase;DHCR7;ortholog	7-DEHYDROCHOLESTEROL REDUCTASE (PTHR21257:SF38)	reductase(PC00198)	0.03902	0.921808
**Q12797**	GBG12_HUMAN	Guanine nucleotide-binding protein G(I)/G(S)/G(O) subunit gamma-12;GNG12;ortholog	GUANINE NUCLEOTIDE-BINDING PROTEIN G(I)/G(S)/G(O) SUBUNIT GAMMA-12 (PTHR13809:SF9)	heterotrimeric G-protein(PC00117)	0.03937	5.053687
**P62241**	DDX21_HUMAN	Nucleolar RNA helicase 2;DDX21;ortholog	NUCLEOLAR RNA HELICASE 2 (PTHR47958:SF109)	NA	0.03971	1.251012
**P00918**	RL24_HUMAN	60S ribosomal protein L24;RPL24;ortholog	60S RIBOSOMAL PROTEIN L24 (PTHR10792:SF44)	ribosomal protein(PC00202)	0.03983	1.16354
**P60953**	MCM3_HUMAN	DNA replication licensing factor MCM3;MCM3;ortholog	DNA REPLICATION LICENSING FACTOR MCM3 (PTHR11630:SF46)	DNA metabolism protein(PC00009)	0.04021	1.238328
**P23434**	VAPB_HUMAN	Vesicle-associated membrane protein-associated protein B/C;VAPB;ortholog	VESICLE-ASSOCIATED MEMBRANE PROTEIN-ASSOCIATED PROTEIN B/C (PTHR10809:SF12)	membrane trafficking regulatory protein(PC00151)	0.04089	0.699019
**P48444**	HXK2_HUMAN	Hexokinase-2;HK2;ortholog	HEXOKINASE-2 (PTHR19443:SF4)	kinase(PC00137)	0.04144	0.875105
**O95292**	PSMD2_HUMAN	26S proteasome non-ATPase regulatory subunit 2;PSMD2;ortholog	26S PROTEASOME NON-ATPASE REGULATORY SUBUNIT 2 (PTHR10943:SF11)	protease(PC00190)	0.04195	2.668984
**Q9NVA2**	SYAC_HUMAN	Alanine--tRNA ligase, cytoplasmic;AARS1;ortholog	ALANINE--TRNA LIGASE, CYTOPLASMIC (PTHR11777:SF36)	aminoacyl-tRNA synthetase(PC00047)	0.04205	0.917524
**Q53GQ0**	TOM70_HUMAN	Mitochondrial import receptor subunit TOM70;TOMM70;ortholog	MITOCHONDRIAL IMPORT RECEPTOR SUBUNIT TOM70 (PTHR46208:SF1)	primary active transporter(PC00068)	0.04411	1.122194
**P45974**	TF_HUMAN	Tissue factor;F3;ortholog	TISSUE FACTOR (PTHR20859:SF22)	transmembrane signal receptor(PC00197)	0.04428	0.756568

**P54136**	SF3A3_HUMAN	Splicing factor 3A subunit 3;SF3A3;ortholog	SPLICING FACTOR 3A SUBUNIT 3 (PTHR12786:SF3)	RNA splicing factor(PC00148)	0.04483	3.710812
**P37837**	PYR1_HUMAN	CAD protein;CAD;ortholog	CAD PROTEIN (PTHR11405:SF5)	ligase(PC00142)	0.04487	1.234783
**P32929**	TR10D_HUMAN	Tumor necrosis factor receptor superfamily member 10D;TNFRSF10D;ortholog	TUMOR NECROSIS FACTOR RECEPTOR SUPERFAMILY MEMBER 10D (PTHR46330:SF10)	transmembrane signal receptor(PC00197)	0.04537	1.101144
**Q15691**	HDGF_HUMAN	Hepatoma-derived growth factor;HDGF;ortholog	HEPATOMA-DERIVED GROWTH FACTOR (PTHR12550:SF41)	transcription cofactor(PC00217)	0.04544	0.953502
**O43615**	S39A7_HUMAN	Zinc transporter SLC39A7;SLC39A7;ortholog	ZINC TRANSPORTER SLC39A7 (PTHR16950:SF25)	primary active transporter(PC00068)	0.04568	0.882531
**P21741**	TOM22_HUMAN	Mitochondrial import receptor subunit TOM22 homolog;TOMM22;ortholog	MITOCHONDRIAL IMPORT RECEPTOR SUBUNIT TOM22 HOMOLOG (PTHR12504:SF3)	primary active transporter(PC00068)	0.04581	0.895941
**P61981**	RHOC_HUMAN	Rho-related GTP-binding protein RhoC;RHOC;ortholog	RHO-RELATED GTP-BINDING PROTEIN RHOC (PTHR24072:SF100)	small GTPase(PC00208)	0.04582	0.870883
**P27708**	UBXN4_HUMAN	UBX domain-containing protein 4;UBXN4;ortholog	UBX DOMAIN-CONTAINING PROTEIN 4 (PTHR46424:SF1)	NA	0.04618	0.959065
**P18085**	SPTB2_HUMAN	Spectrin beta chain, non-erythrocytic 1;SPTBN1;ortholog	SPECTRIN BETA CHAIN, NON-ERYTHROCYTIC 1 (PTHR11915:SF226)	NA	0.0466	0.773288
**Q92597**	MK_HUMAN	Midkine;MDK;ortholog	MIDKINE (PTHR13850:SF2)	growth factor(PC00112)	0.04843	1.167836
**P46777**	GLGB_HUMAN	1,4-alpha-glucan-branching enzyme;GBE1;ortholog	1,4-ALPHA-GLUCAN-BRANCHING ENZYME (PTHR43651:SF3)	amylase(PC00048)	0.04917	1.101776

Table S6. Clinical characteristics of the patients involved in this study.

**Variables**	**Controls (n=22)**	**Cancer recurrence (n=9)**	**ANOVA**
**Age, years, Mean (SD)**	n=22 61.09 (13.15)	n=9 57.89 (21.46)	p= 0.9405
**Pre-Op CA-125, U/mL, Mean (SD)**	n=14 423.60 (460.20)	n=9 645.60 (869.50)	p= 0.5571
**Post-Op CA-125, U/mL, Mean (SD)**	n=13 39.54 (33.01)	n=3 211.70 (197.10)	p= 0.3571
**Post-Chemo CA-125, U/mL, Mean (SD)**	n=11 11.46 (7.69)	n=4 52.43 (82.44)	p= 0.2637
**Serous, n**	16	9	-
**Mucinous, n**	2	-	-
**Clear Cell, n**	1	-	-
**Endometrioid, n**	3	-	-

Clinical characteristics of patient samples. Data are presented as mean (SD). Groups were classified according to patients that were Alive without Disease (AWOD) or patients that presented with Recurrent Disease (RD). (-) Not applicable. Clinical data was not available for all samples.

Table S7. Proteins identified in circulating sEVs isolated from plasma.

ID	Symbol	Entrez Gene Name	Location	Type(s)
**A1BG_HUMAN**	A1BG	alpha-1-B glycoprotein	Extracellular Space	other
**A2MG_HUMAN**	A2M	alpha-2-macroglobulin	Extracellular Space	transporter
**ACES_HUMAN**	ACHE	acetylcholinesterase (Cartwright blood group)	Plasma Membrane	enzyme
**ACTB_HUMAN**	ACTB	actin beta	Cytoplasm	other
**ACTC_HUMAN**	ACTC1	actin alpha cardiac muscle 1	Cytoplasm	enzyme
**ACTN1_HUMAN**	ACTN1	actinin alpha 1	Cytoplasm	transcription regulator
**ADIPO_HUMAN**	ADIPOQ	adiponectin, C1Q and collagen domain containing	Extracellular Space	other
**AFAM_HUMAN**	AFM	afamin	Extracellular Space	transporter
**ANGT_HUMAN**	AGT	angiotensinogen	Extracellular Space	growth factor
**FETUA_HUMAN**	AHSG	alpha 2-HS glycoprotein	Extracellular Space	other
**ALBU_HUMAN**	ALB	albumin	Extracellular Space	transporter
**ALDOA_HUMAN**	ALDOA	aldolase, fructose-bisphosphate A	Cytoplasm	enzyme
**AMBP_HUMAN**	AMBP	alpha-1-microglobulin/bikunin precursor	Extracellular Space	transporter
**ANK1_HUMAN**	ANK1	ankyrin 1	Plasma Membrane	other
**AMPN_HUMAN**	ANPEP	alanyl aminopeptidase, membrane	Plasma Membrane	peptidase
**ANXA2_HUMAN**	ANXA2	annexin A2	Plasma Membrane	other
**SAMP_HUMAN**	APCS	amyloid P component, serum	Extracellular Space	other
**APMAP_HUMAN**	APMAP	adipocyte plasma membrane associated protein	Plasma Membrane	enzyme
**APOA1_HUMAN**	APOA1	apolipoprotein A1	Extracellular Space	transporter
**APOA2_HUMAN**	APOA2	apolipoprotein A2	Extracellular Space	transporter
**APOA4_HUMAN**	APOA4	apolipoprotein A4	Extracellular Space	transporter
**APOB_HUMAN**	APOB	apolipoprotein B	Extracellular Space	transporter
**APOC1_HUMAN**	APOC1	apolipoprotein C1	Extracellular Space	transporter
**APOC2_HUMAN**	APOC2	apolipoprotein C2	Extracellular Space	transporter
**APOC3_HUMAN**	APOC3	apolipoprotein C3	Extracellular Space	transporter

**APOC4_HUMAN**	APOC4	apolipoprotein C4	Extracellular Space	transporter
**APOD_HUMAN**	APOD	apolipoprotein D	Extracellular Space	transporter
**APOE_HUMAN**	APOE	apolipoprotein E	Extracellular Space	transporter
**APOF_HUMAN**	APOF	apolipoprotein F	Extracellular Space	transporter
**APOH_HUMAN**	APOH	apolipoprotein H	Extracellular Space	transporter
**APOL1_HUMAN**	APOL1	apolipoprotein L1	Extracellular Space	transporter
**APOM_HUMAN**	APOM	apolipoprotein M	Plasma Membrane	transporter
**ARGI1_HUMAN**	ARG1	arginase 1	Cytoplasm	enzyme
**ATRN_HUMAN**	ATRN	attractin	Extracellular Space	other
**ZA2G_HUMAN**	AZGP1	alpha-2-glycoprotein 1, zinc-binding	Extracellular Space	transporter
**CHLE_HUMAN**	BCHE	butyrylcholinesterase	Plasma Membrane	enzyme
**BLVRB_HUMAN**	BLVRB	biliverdin reductase B	Cytoplasm	enzyme
**XP32_HUMAN**	C1orf68	chromosome 1 open reading frame 68	Cytoplasm	other
**C1QA_HUMAN**	C1QA	complement C1q A chain	Extracellular Space	other
**C1QB_HUMAN**	C1QB	complement C1q B chain	Extracellular Space	other
**C1QC_HUMAN**	C1QC	complement C1q C chain	Extracellular Space	other
**C1R_HUMAN**	C1R	complement C1r	Extracellular Space	peptidase
**C1S_HUMAN**	C1S	complement C1s	Extracellular Space	peptidase
**CO2_HUMAN**	C2	complement C2	Extracellular Space	peptidase
**CO3_HUMAN**	C3	complement C3	Extracellular Space	peptidase
**CO4A_HUMAN**	C4A/C4B	complement C4A (Rodgers blood group)	Extracellular Space	other
**CO4B_HUMAN**	C4A/C4B	complement C4A (Rodgers blood group)	Extracellular Space	other
**C4BPA_HUMAN**	C4BPA	complement component 4 binding protein alpha	Extracellular Space	other
**CO5_HUMAN**	C5	complement C5	Extracellular Space	cytokine
**CO6_HUMAN**	C6	complement C6	Extracellular Space	other
**CO7_HUMAN**	C7	complement C7	Extracellular Space	other
**CO8A_HUMAN**	C8A	complement C8 alpha chain	Extracellular Space	other
**CO8B_HUMAN**	C8B	complement C8 beta chain	Extracellular Space	other
**CO8G_HUMAN**	C8G	complement C8 gamma chain	Extracellular Space	transporter
**CO9_HUMAN**	C9	complement C9	Extracellular Space	other
**CAH1_HUMAN**	CA1	carbonic anhydrase 1	Cytoplasm	enzyme
**CAH2_HUMAN**	CA2	carbonic anhydrase 2	Cytoplasm	enzyme
**CAMP_HUMAN**	CAMP	cathelicidin antimicrobial peptide	Cytoplasm	other

**CAP1_HUMAN**	CAP1	cyclase associated actin cytoskeleton regulatory protein 1	Plasma Membrane	other
**CASPE_HUMAN**	CASP14	caspase 14	Cytoplasm	peptidase
**CATA_HUMAN**	CAT	catalase	Cytoplasm	enzyme
**CAVN2_HUMAN**	CAVIN2	caveolae associated protein 2	Plasma Membrane	other
**CD36_HUMAN**	CD36	CD36 molecule	Plasma Membrane	transmembrane receptor
**CD59_HUMAN**	CD59	CD59 molecule (CD59 blood group)	Plasma Membrane	other
**CD5L_HUMAN**	CD5L	CD5 molecule like	Plasma Membrane	transmembrane receptor
**CD9_HUMAN**	CD9	CD9 molecule	Plasma Membrane	other
**CDC42_HUMAN**	CDC42	cell division cycle 42	Cytoplasm	enzyme
**CFA46_HUMAN**	CFAP46	cilia and flagella associated protein 46	Cytoplasm	other
**CFAB_HUMAN**	CFB	complement factor B	Extracellular Space	peptidase
**CFAH_HUMAN**	CFH	complement factor H	Extracellular Space	other
**FHR1_HUMAN**	CFHR1	complement factor H related 1	Extracellular Space	other
**FHR4_HUMAN**	CFHR4	complement factor H related 4	Extracellular Space	transporter
**FHR5_HUMAN**	CFHR5	complement factor H related 5	Extracellular Space	other
**CFAI_HUMAN**	CFI	complement factor I	Extracellular Space	peptidase
**COF1_HUMAN**	CFL1	cofilin 1	Nucleus	other
**PROP_HUMAN**	CFP	complement factor properdin	Extracellular Space	other
**CLUS_HUMAN**	CLU	clusterin	Cytoplasm	other
**CO6A3_HUMAN**	COL6A3	collagen type VI alpha 3 chain	Extracellular Space	other
**COL11_HUMAN**	COLEC11	collectin subfamily member 11	Extracellular Space	other
**COMP_HUMAN**	COMP	cartilage oligomeric matrix protein	Extracellular Space	other
**CERU_HUMAN**	CP	ceruloplasmin	Extracellular Space	enzyme
**CBPN_HUMAN**	CPN1	carboxypeptidase N subunit 1	Extracellular Space	peptidase
**CPN2_HUMAN**	CPN2	carboxypeptidase N subunit 2	Extracellular Space	peptidase
**CRP_HUMAN**	CRP	C-reactive protein	Extracellular Space	other
**DOPO_HUMAN**	DBH	dopamine beta-hydroxylase	Cytoplasm	enzyme
**DCD_HUMAN**	DCD	dermcidin	Extracellular Space	other
**DEF3_HUMAN**	DEFA1 (includes others)	defensin alpha 1	Cytoplasm	other
**DSC1_HUMAN**	DSC1	desmocollin 1	Plasma Membrane	other
**DSG1_HUMAN**	DSG1	desmoglein 1	Plasma Membrane	other

**DESP_HUMAN**	DSP	desmoplakin	Plasma Membrane	other
**ECM1_HUMAN**	ECM1	extracellular matrix protein 1	Extracellular Space	transporter
**ENOA_HUMAN**	ENO1	enolase 1	Cytoplasm	enzyme
**41_HUMAN**	EPB41	erythrocyte membrane protein band 4.1	Plasma Membrane	other
**EPB42_HUMAN**	EPB42	erythrocyte membrane protein band 4.2	Plasma Membrane	transporter
**FA10_HUMAN**	F10	coagulation factor X	Extracellular Space	peptidase
**F13A_HUMAN**	F13A1	coagulation factor XIII A chain	Extracellular Space	enzyme
**F13B_HUMAN**	F13B	coagulation factor XIII B chain	Extracellular Space	enzyme
**THRB_HUMAN**	F2	coagulation factor II, thrombin	Extracellular Space	peptidase
**FA5_HUMAN**	F5	coagulation factor V	Extracellular Space	other
**FABP5_HUMAN**	FABP5	fatty acid binding protein 5	Cytoplasm	transporter
**FBLN1_HUMAN**	FBLN1	fibulin 1	Extracellular Space	other
**FCGBP_HUMAN**	FCGBP	Fc fragment of IgG binding protein	Extracellular Space	other
**FCN2_HUMAN**	FCN2	ficolin 2	Extracellular Space	other
**FCN3_HUMAN**	FCN3	ficolin 3	Extracellular Space	other
**URP2_HUMAN**	FERMT3	fermitin family member 3	Cytoplasm	enzyme
**FIBA_HUMAN**	FGA	fibrinogen alpha chain	Extracellular Space	other
**FIBB_HUMAN**	FGB	fibrinogen beta chain	Extracellular Space	other
**FIBG_HUMAN**	FGG	fibrinogen gamma chain	Extracellular Space	other
**FILA_HUMAN**	FLG	filaggrin	Cytoplasm	other
**FILA2_HUMAN**	FLG2	filaggrin family member 2	Cytoplasm	other
**FLNA_HUMAN**	FLNA	filamin A	Cytoplasm	other
**FINC_HUMAN**	FN1	fibronectin 1	Extracellular Space	enzyme
**G3P_HUMAN**	GAPDH	glyceraldehyde-3-phosphate dehydrogenase	Cytoplasm	enzyme
**VTDB_HUMAN**	GC	GC vitamin D binding protein	Extracellular Space	transporter
**GNAI2_HUMAN**	GNAI2	G protein subunit alpha i2	Plasma Membrane	enzyme
**GP1BA_HUMAN**	GP1BA	glycoprotein Ib platelet subunit alpha	Plasma Membrane	transmembrane receptor
**GP1BB_HUMAN**	GP1BB	glycoprotein Ib platelet subunit beta	Plasma Membrane	other
**GPIX_HUMAN**	GP9	glycoprotein IX platelet	Plasma Membrane	other
**PHLD_HUMAN**	GPLD1	glycosylphosphatidylinositol specific phospholipase D1	Cytoplasm	enzyme
**GELS_HUMAN**	GSN	gelsolin	Extracellular Space	other
**GLPA_HUMAN**	GYPA	glycophorin A (MNS blood group)	Plasma Membrane	other

**GLPC_HUMAN**	GYPC	glycophorin C (Gerbich blood group)	Plasma Membrane	other
**H2AJ_HUMAN**	H2AJ	H2A.J histone	Cytoplasm	other
**H2B2F_HUMAN**	H2BC18	H2B clustered histone 18	Nucleus	other
**HBB_HUMAN**	HBB	hemoglobin subunit beta	Cytoplasm	transporter
**HBD_HUMAN**	HBD	hemoglobin subunit delta	Other	transporter
**HBG2_HUMAN**	HBG2	hemoglobin subunit gamma 2	Cytoplasm	other
**HPT_HUMAN**	HP	haptoglobin	Extracellular Space	peptidase
**HPTR_HUMAN**	HPR	haptoglobin-related protein	Extracellular Space	peptidase
**HEMO_HUMAN**	HPX	hemopexin	Extracellular Space	transporter
**HRG_HUMAN**	HRG	histidine rich glycoprotein	Extracellular Space	other
**HORN_HUMAN**	HRNR	hornerin	Cytoplasm	other
**HSP7C_HUMAN**	HSPA8	heat shock protein family A (Hsp70) member 8	Cytoplasm	enzyme
**ALS_HUMAN**	IGFALS	insulin like growth factor binding protein acid labile subunit	Extracellular Space	other
**IGHA1_HUMAN**	IGHA1	immunoglobulin heavy constant alpha 1	Extracellular Space	other
**IGHA2_HUMAN**	IGHA2	immunoglobulin heavy constant alpha 2 (A2m marker)	Extracellular Space	other
**IGHD_HUMAN**	IGHD	immunoglobulin heavy constant delta	Extracellular Space	other
**IGHG1_HUMAN**	IGHG1	immunoglobulin heavy constant gamma 1 (G1m marker)	Extracellular Space	other
**IGHG2_HUMAN**	IGHG2	immunoglobulin heavy constant gamma 2 (G2m marker)	Plasma Membrane	other
**IGHG3_HUMAN**	IGHG3	immunoglobulin heavy constant gamma 3 (G3m marker)	Extracellular Space	other
**IGHG4_HUMAN**	IGHG4	immunoglobulin heavy constant gamma 4 (G4m marker)	Extracellular Space	other
**IGHM_HUMAN**	IGHM	immunoglobulin heavy constant mu	Plasma Membrane	transmembrane receptor
**HV118_HUMAN**	IGHV1-18	immunoglobulin heavy variable 1-18	Other	other
**HV124_HUMAN**	IGHV1-24	immunoglobulin heavy variable 1-24	Other	other
**HV103_HUMAN**	IGHV1-3	immunoglobulin heavy variable 1-3	Other	other
**HV146_HUMAN**	IGHV1-46	immunoglobulin heavy variable 1-46	Other	other
**HV158_HUMAN**	IGHV1-58	immunoglobulin heavy variable 1-58	Other	other
**HV692_HUMAN**	IGHV1-69-2	immunoglobulin heavy variable 1-69-2	Other	other
**HV69D_HUMAN**	IGHV1-69D	immunoglobulin heavy variable 1-69D	Other	other
**HV226_HUMAN**	IGHV2-26	immunoglobulin heavy variable 2-26	Other	other
**HV205_HUMAN**	IGHV2-5	immunoglobulin heavy variable 2-5	Other	other

**HV70D_HUMAN**	IGHV2-70D	immunoglobulin heavy variable 2-70D	Other	other
**HV315_HUMAN**	IGHV3-15	immunoglobulin heavy variable 3-15	Other	other
**HV320_HUMAN**	IGHV3-20	immunoglobulin heavy variable 3-20	Other	other
**HV323_HUMAN**	IGHV3-23	immunoglobulin heavy variable 3-23	Extracellular Space	other
**HV335_HUMAN**	IGHV3-35	immunoglobulin heavy variable 3-35 (non-functional)	Other	other
**HV349_HUMAN**	IGHV3-49	immunoglobulin heavy variable 3-49	Other	other
**HV364_HUMAN**	IGHV3-64	immunoglobulin heavy variable 3-64	Other	other
**HV64D_HUMAN**	IGHV3-64D	immunoglobulin heavy variable 3-64D	Other	other
**HV366_HUMAN**	IGHV3-66	immunoglobulin heavy variable 3-66	Other	other
**HV307_HUMAN**	IGHV3-7	immunoglobulin heavy variable 3-7	Extracellular Space	other
**HV372_HUMAN**	IGHV3-72	immunoglobulin heavy variable 3-72	Other	other
**HV373_HUMAN**	IGHV3-73	immunoglobulin heavy variable 3-73	Other	other
**HV374_HUMAN**	IGHV3-74	immunoglobulin heavy variable 3-74	Other	other
**HV434_HUMAN**	IGHV4-34	immunoglobulin heavy variable 4-34	Other	other
**HV5X1_HUMAN**	IGHV5-10-1	immunoglobulin heavy variable 5-10-1	Other	other
**HV551_HUMAN**	IGHV5-51	immunoglobulin heavy variable 5-51	Other	other
**HV601_HUMAN**	IGHV6-1	immunoglobulin heavy variable 6-1	Other	other
**IGKC_HUMAN**	IGKC	immunoglobulin kappa constant	Extracellular Space	other
**KVD16_HUMAN**	IGKC	immunoglobulin kappa constant	Extracellular Space	other
**KV113_HUMAN**	IGKV1-13	immunoglobulin kappa variable 1-13 (gene/pseudogene)	Other	other
**KV106_HUMAN**	IGKV1-6	immunoglobulin kappa variable 1-6	Other	other
**KV133_HUMAN**	IGKV1D-33	immunoglobulin kappa variable 1D-33	Extracellular Space	other
**KVD08_HUMAN**	IGKV1D-8	immunoglobulin kappa variable 1D-8	Other	other
**KV224_HUMAN**	IGKV2-24	immunoglobulin kappa variable 2-24	Other	other
**KV230_HUMAN**	IGKV2-30	immunoglobulin kappa variable 2-30	Extracellular Space	other
**KVD40_HUMAN**	IGKV2-40	immunoglobulin kappa variable 2-40	Extracellular Space	other
**KVD29_HUMAN**	IGKV2D-29	immunoglobulin kappa variable 2D-29	Other	other
**KV320_HUMAN**	IGKV3-20	immunoglobulin kappa variable 3-20	Extracellular Space	other
**KV311_HUMAN**	IGKV3D-11	immunoglobulin kappa variable 3D-11	Extracellular Space	other
**KVD15_HUMAN**	IGKV3D-15	immunoglobulin kappa variable 3D-15 (gene/pseudogene)	Other	other
**KVD20_HUMAN**	IGKV3D-20	immunoglobulin kappa variable 3D-20	Extracellular Space	other

**KVD07_HUMAN**	IGKV3D-7	immunoglobulin kappa variable 3D-7	Other	other
**KV401_HUMAN**	IGKV4-1	immunoglobulin kappa variable 4-1	Extracellular Space	other
**KVD21_HUMAN**	IGKV6D-21	immunoglobulin kappa variable 6D-21 (non-functional)	Other	other
**IGLC3_HUMAN**	IGLC3	immunoglobulin lambda constant 3 (Kern-Oz+ marker)	Extracellular Space	other
**IGLC7_HUMAN**	IGLC7	immunoglobulin lambda constant 7	Extracellular Space	other
**IGLL5_HUMAN**	IGLL1/IGLL5	immunoglobulin lambda like polypeptide 1	Plasma Membrane	other
**LV136_HUMAN**	IGLV1-36	immunoglobulin lambda variable 1-36	Other	other
**LV140_HUMAN**	IGLV1-40	immunoglobulin lambda variable 1-40	Other	other
**LV147_HUMAN**	IGLV1-47	immunoglobulin lambda variable 1-47	Extracellular Space	other
**LV151_HUMAN**	IGLV1-51	immunoglobulin lambda variable 1-51	Extracellular Space	other
**LVX54_HUMAN**	IGLV10-54	immunoglobulin lambda variable 10-54	Other	other
**LV211_HUMAN**	IGLV2-11	immunoglobulin lambda variable 2-11	Extracellular Space	other
**LV218_HUMAN**	IGLV2-18	immunoglobulin lambda variable 2-18	Other	other
**LV310_HUMAN**	IGLV3-10	immunoglobulin lambda variable 3-10	Other	other
**LV319_HUMAN**	IGLV3-19	immunoglobulin lambda variable 3-19	Extracellular Space	other
**LV321_HUMAN**	IGLV3-21	immunoglobulin lambda variable 3-21	Extracellular Space	other
**LV327_HUMAN**	IGLV3-27	immunoglobulin lambda variable 3-27	Other	other
**LV469_HUMAN**	IGLV4-69	immunoglobulin lambda variable 4-69	Other	other
**LV657_HUMAN**	IGLV6-57	immunoglobulin lambda variable 6-57	Extracellular Space	other
**LV746_HUMAN**	IGLV7-46	immunoglobulin lambda variable 7-46 (gene/pseudogene)	Other	other
**LV861_HUMAN**	IGLV8-61	immunoglobulin lambda variable 8-61	Other	other
**LV949_HUMAN**	IGLV9-49	immunoglobulin lambda variable 9-49	Other	other
**ITA2B_HUMAN**	ITGA2B	integrin subunit alpha 2b	Plasma Membrane	transmembrane receptor
**ITB1_HUMAN**	ITGB1	integrin subunit beta 1	Plasma Membrane	transmembrane receptor
**ITB3_HUMAN**	ITGB3	integrin subunit beta 3	Plasma Membrane	transmembrane receptor
**ITIH1_HUMAN**	ITIH1	inter-alpha-trypsin inhibitor heavy chain 1	Extracellular Space	other
**ITIH2_HUMAN**	ITIH2	inter-alpha-trypsin inhibitor heavy chain 2	Extracellular Space	other
**ITIH3_HUMAN**	ITIH3	inter-alpha-trypsin inhibitor heavy chain 3	Extracellular Space	other
**ITIH4_HUMAN**	ITIH4	inter-alpha-trypsin inhibitor heavy chain 4	Extracellular Space	other
**IGJ_HUMAN**	JCHAIN	joining chain of multimeric IgA and IgM	Extracellular Space	other
**PLAK_HUMAN**	JUP	junction plakoglobin	Plasma Membrane	other

**KLKB1_HUMAN**	KLKB1	kallikrein B1	Extracellular Space	peptidase
**KNG1_HUMAN**	KNG1	kininogen 1	Extracellular Space	other
**KNG1_HUMAN**	KNG1	kininogen 1	Extracellular Space	other
**KPRP_HUMAN**	KPRP	keratinocyte proline rich protein	Cytoplasm	other
**K2C1_HUMAN**	KRT1	keratin 1	Cytoplasm	other
**K1C10_HUMAN**	KRT10	keratin 10	Cytoplasm	other
**K1C14_HUMAN**	KRT14	keratin 14	Cytoplasm	other
**K1C16_HUMAN**	KRT16	keratin 16	Cytoplasm	other
**K1C17_HUMAN**	KRT17	keratin 17	Cytoplasm	other
**K22E_HUMAN**	KRT2	keratin 2	Cytoplasm	other
**K1H1_HUMAN**	KRT31	keratin 31	Cytoplasm	other
**KT33A_HUMAN**	KRT33A	keratin 33A	Extracellular Space	other
**KT33B_HUMAN**	KRT33B	keratin 33B	Extracellular Space	other
**KRT34_HUMAN**	KRT34	keratin 34	Cytoplasm	other
**KRT35_HUMAN**	KRT35	keratin 35	Cytoplasm	other
**KRT36_HUMAN**	KRT36	keratin 36	Cytoplasm	other
**K2C4_HUMAN**	KRT4	keratin 4	Cytoplasm	other
**K2C5_HUMAN**	KRT5	keratin 5	Cytoplasm	other
**K2C6B_HUMAN**	KRT6B	keratin 6B	Cytoplasm	other
**K2C73_HUMAN**	KRT73	keratin 73	Extracellular Space	other
**K2C1B_HUMAN**	KRT77	keratin 77	Cytoplasm	other
**K2C78_HUMAN**	KRT78	keratin 78	Cytoplasm	other
**K2C80_HUMAN**	KRT80	keratin 80	Cytoplasm	other
**KRT82_HUMAN**	KRT82	keratin 82	Cytoplasm	other
**KRT83_HUMAN**	KRT83	keratin 83	Cytoplasm	other
**KRT85_HUMAN**	KRT85	keratin 85	Extracellular Space	other
**KRT86_HUMAN**	KRT86	keratin 86	Cytoplasm	other
**KR87P_HUMAN**	KRT87P	keratin 87 pseudogene	Extracellular Space	other
**K1C9_HUMAN**	KRT9	keratin 9	Cytoplasm	other
**KRA21_HUMAN**	KRTAP2-1	keratin associated protein 2-1	Cytoplasm	other
**LAMB1_HUMAN**	LAMB1	laminin subunit beta 1	Extracellular Space	other
**LBP_HUMAN**	LBP	lipopolysaccharide binding protein	Plasma Membrane	transporter
**LBX2_HUMAN**	LBX2	ladybird homeobox 2	Nucleus	transcription regulator

**LDHB_HUMAN**	LDHB	lactate dehydrogenase B	Cytoplasm	enzyme
**LG3BP_HUMAN**	LGALS3BP	galectin 3 binding protein	Plasma Membrane	transmembrane receptor
**APOA_HUMAN**	LPA	lipoprotein(a)	Extracellular Space	other
**A2GL_HUMAN**	LRG1	leucine rich alpha-2-glycoprotein 1	Extracellular Space	other
**TRFL_HUMAN**	LTF	lactotransferrin	Extracellular Space	peptidase
**LUM_HUMAN**	LUM	lumican	Extracellular Space	other
**LYSC_HUMAN**	LYZ	lysozyme	Extracellular Space	enzyme
**MASP1_HUMAN**	MASP1	mannan binding lectin serine peptidase 1	Extracellular Space	peptidase
**MASP1_HUMAN**	MASP1	mannan binding lectin serine peptidase 1	Extracellular Space	peptidase
**MBL2_HUMAN**	MBL2	mannose binding lectin 2	Extracellular Space	other
**MMRN1_HUMAN**	MMRN1	multimerin 1	Extracellular Space	other
**PERM_HUMAN**	MPO	myeloperoxidase	Cytoplasm	enzyme
**EM55_HUMAN**	MPP1	membrane palmitoylated protein 1	Plasma Membrane	kinase
**MUC1_HUMAN**	MUC1	mucin 1, cell surface associated	Plasma Membrane	other
**MUC16_HUMAN**	MUC16	mucin 16, cell surface associated	Cytoplasm	other
**MUC5A_HUMAN**	MUC5AC	mucin 5AC, oligomeric mucus/gel-forming	Cytoplasm	peptidase
**MUC5B_HUMAN**	MUC5B	mucin 5B, oligomeric mucus/gel-forming	Extracellular Space	peptidase
**MYH9_HUMAN**	MYH9	myosin heavy chain 9	Cytoplasm	enzyme
**NOP53_HUMAN**	NOP53	NOP53 ribosome biogenesis factor	Cytoplasm	other
**A1AG1_HUMAN**	ORM1	orosomucoid 1	Extracellular Space	other
**A1AG2_HUMAN**	ORM2	orosomucoid 2	Extracellular Space	other
**PDIA1_HUMAN**	P4HB	prolyl 4-hydroxylase subunit beta	Cytoplasm	enzyme
**PCYOX_HUMAN**	PCYOX1	prenylcysteine oxidase 1	Cytoplasm	enzyme
**PECA1_HUMAN**	PECAM1	platelet and endothelial cell adhesion molecule 1	Plasma Membrane	other
**PLF4_HUMAN**	PF4	platelet factor 4	Extracellular Space	cytokine
**PROF1_HUMAN**	PFN1	profilin 1	Cytoplasm	other
**PGRP2_HUMAN**	PGLYRP2	peptidoglycan recognition protein 2	Plasma Membrane	transmembrane receptor
**PI16_HUMAN**	PI16	peptidase inhibitor 16	Extracellular Space	other
**PIGR_HUMAN**	PIGR	polymeric immunoglobulin receptor	Plasma Membrane	transporter
**PIP_HUMAN**	PIP	prolactin induced protein	Extracellular Space	peptidase
**KPYM_HUMAN**	PKM	pyruvate kinase M1/2	Cytoplasm	kinase
**PKP1_HUMAN**	PKP1	plakophilin 1	Plasma Membrane	other
**PLEK_HUMAN**	PLEK	pleckstrin	Cytoplasm	other

**PLMN_HUMAN**	PLG	plasminogen	Extracellular Space	peptidase
**PLTP_HUMAN**	PLTP	phospholipid transfer protein	Extracellular Space	enzyme
**POF1B_HUMAN**	POF1B	POF1B actin binding protein	Plasma Membrane	other
**PON1_HUMAN**	PON1	paraoxonase 1	Extracellular Space	phosphatase
**PPIA_HUMAN**	PPIA	peptidylprolyl isomerase A	Cytoplasm	enzyme
**PRDX2_HUMAN**	PRDX2	peroxiredoxin 2	Cytoplasm	enzyme
**PRDX6_HUMAN**	PRDX6	peroxiredoxin 6	Cytoplasm	enzyme
**PRG4_HUMAN**	PRG4	proteoglycan 4	Extracellular Space	other
**PROS_HUMAN**	PROS1	protein S	Extracellular Space	other
**PZP_HUMAN**	PZP	PZP alpha-2-macroglobulin like	Extracellular Space	other
**QSOX1_HUMAN**	QSOX1	quiescin sulfhydryl oxidase 1	Cytoplasm	enzyme
**RAB1B_HUMAN**	RAB1B	RAB1B, member RAS oncogene family	Cytoplasm	other
**RAP1B_HUMAN**	RAP1B	RAP1B, member of RAS oncogene family	Cytoplasm	enzyme
**RAP2B_HUMAN**	RAP2B	RAP2B, member of RAS oncogene family	Plasma Membrane	enzyme
**RHAG_HUMAN**	RHAG	Rh associated glycoprotein	Plasma Membrane	peptidase
**RHD_HUMAN**	RHCE/RHD	Rh blood group D antigen	Plasma Membrane	transporter
**RS27A_HUMAN**	RPS27A	ribosomal protein S27a	Cytoplasm	other
**S10A8_HUMAN**	S100A8	S100 calcium binding protein A8	Cytoplasm	other
**S10A9_HUMAN**	S100A9	S100 calcium binding protein A9	Cytoplasm	other
**SAA1_HUMAN**	SAA1	serum amyloid A1	Extracellular Space	transporter
**SAA2_HUMAN**	SAA2	serum amyloid A2	Extracellular Space	other
**SAA4_HUMAN**	SAA4	serum amyloid A4, constitutive	Extracellular Space	transporter
**A1AT_HUMAN**	SERPINA1	serpin family A member 1	Extracellular Space	other
**ZPI_HUMAN**	SERPINA10	serpin family A member 10	Extracellular Space	other
**AACT_HUMAN**	SERPINA3	serpin family A member 3	Extracellular Space	other
**CBG_HUMAN**	SERPINA6	serpin family A member 6	Extracellular Space	other
**SPB12_HUMAN**	SERPINB12	serpin family B member 12	Cytoplasm	other
**ANT3_HUMAN**	SERPINC1	serpin family C member 1	Extracellular Space	enzyme
**HEP2_HUMAN**	SERPIND1	serpin family D member 1	Extracellular Space	other
**PEDF_HUMAN**	SERPINF1	serpin family F member 1	Extracellular Space	other
**A2AP_HUMAN**	SERPINF2	serpin family F member 2	Extracellular Space	other
**IC1_HUMAN**	SERPING1	serpin family G member 1	Extracellular Space	other
**SHBG_HUMAN**	SHBG	sex hormone binding globulin	Extracellular Space	other

**S29A1_HUMAN**	SLC29A1	solute carrier family 29 member 1 (Augustine blood group)	Plasma Membrane	transporter
**GTR1_HUMAN**	SLC2A1	solute carrier family 2 member 1	Plasma Membrane	transporter
**GTR14_HUMAN**	SLC2A14	solute carrier family 2 member 14	Nucleus	transporter
**B3AT_HUMAN**	SLC4A1	solute carrier family 4 member 1 (Diego blood group)	Plasma Membrane	transporter
**SPR2G_HUMAN**	SPRR2G	small proline rich protein 2G	Cytoplasm	other
**SPTA1_HUMAN**	SPTA1	spectrin alpha, erythrocytic 1	Cytoplasm	other
**SPTB1_HUMAN**	SPTB	spectrin beta, erythrocytic	Plasma Membrane	other
**SRGN_HUMAN**	SRGN	serglycin	Cytoplasm	other
**SRCRL_HUMAN**	SSC5D	scavenger receptor cysteine rich family member with 5 domains	Plasma Membrane	transmembrane receptor
**STOM_HUMAN**	STOM	stomatin	Plasma Membrane	other
**SYNE2_HUMAN**	SYNE2	spectrin repeat containing nuclear envelope protein 2	Nucleus	other
**TAGL2_HUMAN**	TAGLN2	transgelin 2	Cytoplasm	other
**TRFE_HUMAN**	TF	transferrin	Extracellular Space	transporter
**TFR1_HUMAN**	TFRC	transferrin receptor	Plasma Membrane	transporter
**BGH3_HUMAN**	TGFBI	transforming growth factor beta induced	Extracellular Space	other
**TGM3_HUMAN**	TGM3	transglutaminase 3	Cytoplasm	enzyme
**TSP1_HUMAN**	THBS1	thrombospondin 1	Extracellular Space	other
**TLN1_HUMAN**	TLN1	talin 1	Plasma Membrane	other
**TENA_HUMAN**	TNC	tenascin C	Extracellular Space	other
**TSN1_HUMAN**	TSPAN1	tetraspanin 1	Cytoplasm	other
**TTHY_HUMAN**	TTR	transthyretin	Extracellular Space	transporter
**TBA1B_HUMAN**	TUBA1B	tubulin alpha 1b	Cytoplasm	other
**TBA4A_HUMAN**	TUBA4A	tubulin alpha 4a	Cytoplasm	other
**TBB5_HUMAN**	TUBB	tubulin beta class I	Cytoplasm	other
**TBB1_HUMAN**	TUBB1	tubulin beta 1 class VI	Cytoplasm	other
**VCAM1_HUMAN**	VCAM1	vascular cell adhesion molecule 1	Plasma Membrane	transmembrane receptor
**VINC_HUMAN**	VCL	vinculin	Plasma Membrane	enzyme
**TERA_HUMAN**	VCP	valosin containing protein	Cytoplasm	enzyme
**VIME_HUMAN**	VIM	vimentin	Cytoplasm	other
**VTNC_HUMAN**	VTN	vitronectin	Extracellular Space	other
**VWF_HUMAN**	VWF	von Willebrand factor	Extracellular Space	other

**1433E_HUMAN**	YWHAE	tyrosine 3-monooxygenase/tryptophan 5-monooxygenase activation protein epsilon	Cytoplasm	other
**1433G_HUMAN**	YWHAG	tyrosine 3-monooxygenase/tryptophan 5-monooxygenase activation protein gamma	Cytoplasm	other
**1433F_HUMAN**	YWHAH	tyrosine 3-monooxygenase/tryptophan 5-monooxygenase activation protein eta	Cytoplasm	transcription regulator
**1433Z_HUMAN**	YWHAZ	tyrosine 3-monooxygenase/tryptophan 5-monooxygenase activation protein zeta	Cytoplasm	enzyme
**ZFHX2_HUMAN**	ZFHX2	zinc finger homeobox 2	Nucleus	transcription regulator

Table S8. Peptide separation was performed at a total flow rate of 5 µL/mL according to the gradient conditions listed below.

Time (min)	Mobile phase A %	Mobile phase B %
0	97	3
15	60	40
18	5	95
22	5	95
25	97	3
30	97	3

Table S9. Chromatography was performed with solvent A (100% H_2O, 0.1% FA) and solvent B (100% ACN, 0.1% FA) at a total flow rate of 15 µL/mL according to the gradient conditions listed below.

Time (min)	Mobile phase A %	Mobile phase B %
0	95	5
1	90	10
8.5	60	40
9.5	10	90
11.5	10	90
12	95	5
18	95	5

A

CD9– around 30 KDa

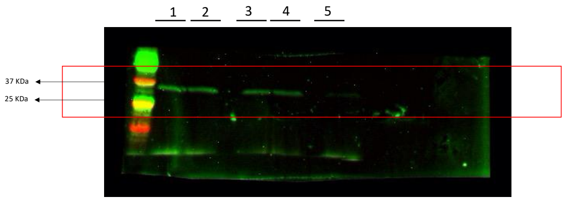

1 and 2 = 1% O_2 sEVs
3 and 4 = 8% O_2 sEVs
5 = cells

B

TSG101– around 45 KDa

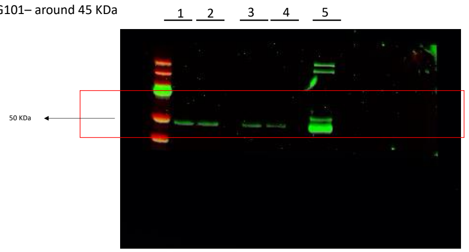

1 and 2 = 1% O_2 sEVs
3 and 4 = 8% O_2 sEVs
5 = cells

C

GRP94 – around 100 KDa

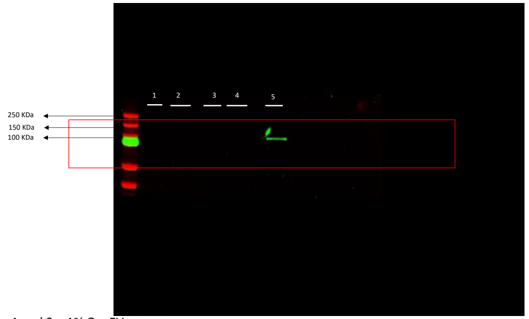

1 and 2 = 1% O_2 sEVs
3 and 4 = 8% O_2 sEVs
5 = cells

Figure S4. Original Western blots used in Figure 3B. A) CD9, B) TSG101, and C) GRP94.
